# A Mechanochemical Reaction Cascade for Controlling Load‐Strengthening of a Mechanochromic Polymer

**DOI:** 10.1002/anie.202010043

**Published:** 2020-09-25

**Authors:** Yifei Pan, Huan Zhang, Piaoxue Xu, Yancong Tian, Chenxu Wang, Shishuai Xiang, Roman Boulatov, Wengui Weng

**Affiliations:** ^1^ Department of Chemistry College of Chemistry and Chemical Engineering Xiamen University 422 South Siming Road Xiamen Fujian 361005 P. R. China; ^2^ Department of Chemistry University of Liverpool and Donnan Lab G31, Crown St. Liverpool L69 7ZD UK

**Keywords:** crosslinking, maleimide, mechanochromism, mechanophores, spirothiopyran

## Abstract

We demonstrate an intermolecular reaction cascade to control the force which triggers crosslinking of a mechanochromic polymer of spirothiopyran (STP). Mechanochromism arises from rapid reversible force‐sensitive isomerization of STP to a merocyanine, which reacts rapidly with activated C=C bonds. The concentration of such bonds, and hence the crosslinking rate, is controlled by force‐dependent dissociation of a Diels–Alder adduct of anthracene and maleimide. Because the adduct requires ca. 1 nN higher force to dissociate at the same rate as that of STP isomerization, the cascade limits crosslinking to overstressed regions of the material, which are at the highest rate of material damage. Using comb polymers decreased the minimum concentration of mechanophores required to crosslinking by about 100‐fold compared to previous examples of load‐strengthening materials. The approach described has potential for controlling a broad range of reaction sequences triggered by mechanical load.

## Graphical abstract

A mechanochemical reaction cascade for controlling load‐strengthening of a mechanochromic polymer is realized by using two comb polymers, each containing either spirothiopyran or a Diels–Alder adduct in the proximity of the branch points. The threshold force to trigger crosslinking is increased by ca. 1 nN and the minimum concentration of mechanophores required for crosslinking is decreased by 100‐fold compared to previous examples.

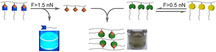

A major aspiration of contemporary polymer mechanochemistry is to reproduce aspects of the sophisticated response of living tissue to mechanical load in synthetic materials.^[1]^ Both types of materials degrade under mechanical load, but only the living tissue can sense and signal this damage (for example, as pain), and trigger its repairs.[^2^, ^3^, ^4^]

Damage‐signaling in synthetic materials is generally achieved by exploiting mechanochromism, or mechanically triggered changes in the absorption or emission properties of a material. Numerous mechanochromic polymers are known,[^5^, ^6^] including those containing spiro‐ or benzopyrans,[^7^, ^8^, ^9^] rhodamine,^[10]^ dimers of stable radicals,^[11]^ Diels–Alder adducts,[^12^, ^13^] cinnamate,[^14^, ^15^] coumarin,^[16]^ and anthracene^[17]^ dimers, metal complexes,^[18]^ dioxetanes,[^19^, ^20^, ^21^, ^22^] energy transfer systems,[^23^, ^24^] and an indene derivative.^[25]^

The closest that synthetic polymers have come to autonomic self‐repair are load‐induced crosslinking or polymerization. To date, the former was demonstrated in four separate polymers each comprised of two types of monomers: one whose isomerization was accelerated by mechanical load, and the other that reacted spontaneously with the isomerization product to form the crosslinks.[^26^, ^27^, ^28^, ^29^] Load‐induced polymerization was achieved when mechanical load generated a catalyst or initiator for polymerization of monomers dissolved in the material prior to loading.[^30^, ^31^, ^32^, ^33^, ^34^]

Endowing polymers with both damage‐signaling and self‐reinforcing capacity requires either increasing the number of distinct components, which presents both synthetic challenges and the need to avoid cross‐reactivity in a loaded material or creating dual mechanophores. These are reactive sites that are both mechanochromic and either initiate or catalyze polymerization of the dissolved monomers, or react stoichiometrically with complementary sites on adjacent chains for crosslinking. Only the latter approach was demonstrated to date,[^26^, ^31^] albeit at a cost of impractically low threshold force for crosslinking onset. In other words, in the only two examples of polymers that are both damage‐signaling and self‐reinforcing,[^26^, ^31^] crosslinking likely occurs at a load that is too low to cause significant material damage.

Although it is not yet known how to design monomers in which desired chemistry is triggered by a load of any desired magnitude, mechanochemical reaction cascades[^35^, ^36^, ^37^, ^38^] offer a general strategy of decoupling the chemistry from the triggering force by using a sacrificial molecular moiety. The sacrificial moiety blocks the desired chemistry until it itself reacts mechanochemically by sequestering (or masking) a reactant needed for the desired chemistry to occur. The force necessary to affect the sacrificial reaction is independent of the mechanochemical kinetics of the target reaction. As long as the sacrificial reaction requires higher force than the target reaction, the kinetics is determined by the sacrificial reaction, while the target reaction delivers the desired chemistry. Mechanochemical cascades that increased threshold force of a target reaction are limited to a single intramolecular example.^[37]^

Here we describe an intermolecular reaction cascade that increases the threshold force which triggers crosslinking of a mechanochromic polymer by >1 nN. This cascade comprises three reactions (Scheme 1): force‐dependent isomerization of spirothiopyran, STP, to thiomerocyanine (TMC), which is responsible for mechanochromism; spontaneous addition of TMC to the C=C bond of maleimide, which crosslinks the material, and force‐dependent dissociation of an anthracene/maleimide Diels–Alder adduct, DA, which controls the force that triggers this crosslinking. Computational and experimental evidence reported below indicate that at low load (single‐chain force <0.5 nN) the material is inert on practical timescales; at moderate loads (ca. 0.5 to ca. 1.5 nN) the stress response is reversible and exclusively mechanochromic; at high loads the material crosslinks irreversibly. Masking a component of the crosslinking pair also eliminates undesired light‐ and heat‐induced crosslinking, and introduce a second mechanochromic reaction, thanks to anthracene absorption at 260–400 nm and fluorescence at 375–500 nm.^[17]^

**Scheme 1:**
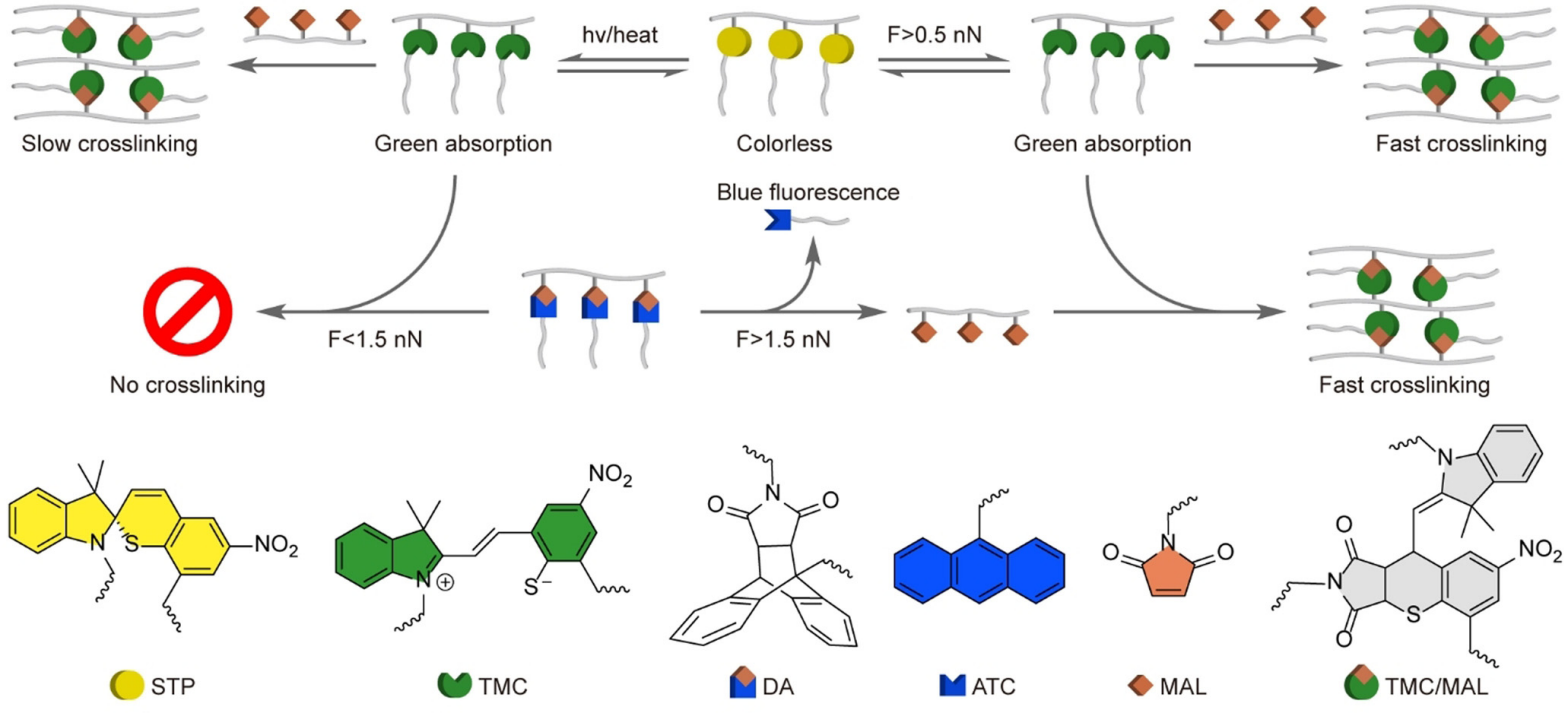
An intermolecular mechanochemical reaction cascade for controlling load induced crosslinking of a mechanochromic material. In this implementation, spirothiopyran (STP, yellow) and anthracene/maleimide adduct (DA, white) are located in long side chains of comb polymers. STP is in a rapid equilibrium with a green merocyanine form (TMC, green), which reacts spontaneously with activated C=C bonds, such as those in maleimide (MAL, orange). The position of the equilibrium is affected by temperature, light, and mechanical load, with the latter favoring the TMC form. A polymer containing both STP and maleimide crosslinks slowly in the dark and rapidly under irradiation or modest mechanical load. Masking maleimide as a mechanochemically labile adduct with anthracene (ATC, blue) eliminates crosslinking in the absence of load and increases the onset force of mechanochemical crosslinking by 1 nN because the adduct is inert unless is stretched to force >1.5 nN. At this force, rapid dissociation of the adduct releases maleimide, enabling fast crosslinking.

Quantum‐chemical calculations of force‐dependent activation free energies of STP isomerization and the anthracene/maleimide adduct dissociation (Figure 1) explain the rate‐determining role of the latter reaction in the load‐induced crosslinking cascade. Calculations at the CAM‐B3LYP/6–311+G(d) level of DFT with THF‐parameterized CPCM model of the reaction solvent suggest that mechanochemical dissociation of the DA adduct used in our study follows the same mechanism as previously reported for other anthracene/maleimide derivatives.[^39^, ^40^] The dissociation is concerted up to 0.5 nN of force, above which a two‐step mechanism traversing two open‐shell singlet transition states dominates (Figure 1; Supporting Information, Figure S1). As expected,^[41]^ the bridgehead, C9‐bound CH_2_ group increases the thermal stability of the DA adduct compared to C9‐alkoxy derivatives studied previously,^[5]^ but does not change the sensitivity of the reaction to force. The mechanism of isomerization of STP has not been reported and is likely complex.^[42]^ Extrapolating from the assumed mechanism of spiropyran isomerization,[^43^, ^44^] its activation free energy, Δ*G* ^≠^_f_ (green curve, Figure 1) is calculated to be about 10 kcal mol^−1^ lower than that for the DA dissociation (blue curve, Figure 1) at the same force of up to 2 nN. The rate of bimolecular addition of TMC to maleimide (gray line, Figure 1) is force independent and faster than DA dissociation (blue curve, Figure 1) at <2 nN (depending on the steady‐state concentration of the two reactants). Consequently, up to this force the kinetics of crosslinking is determined by the rate of mechanochemical dissociation of the DA adduct, whereas at higher force, the kinetics of TMC/maleimide addition becomes rate‐determining. The circa 10 kcal mol^−1^ difference of the two force‐dependent Δ*G* ^≠^_f_ means that using the DA adduct as the source of maleimide increases the minimum force at which crosslinking occurs at practical rate by about 1 nN.

**Figure 1:**
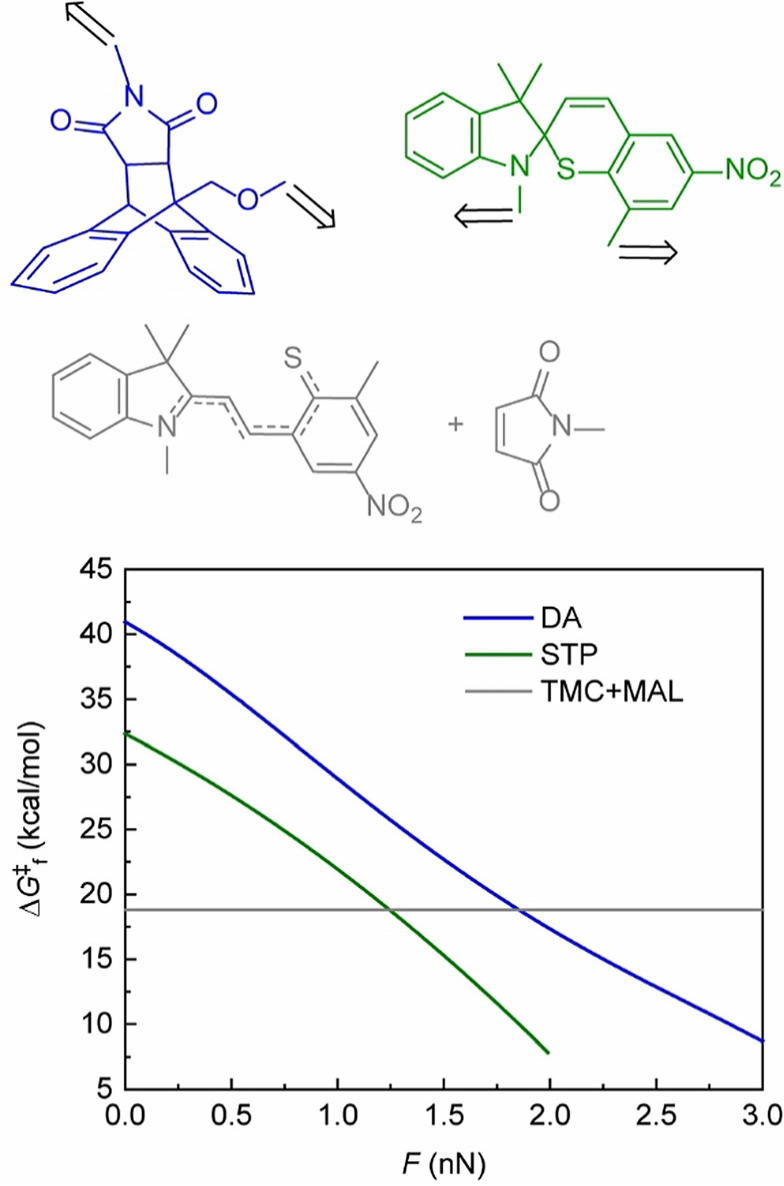
The standard activation free energy of anthracene/maleimide DA adduct dissociation, STP isomerization and the addition of TMC and maleimide at CAM‐B3LYP/6–311+G(d) in THF‐parameterized CPCM and the molecular structures of the reactants used. The pulling axis is shown by the arrows. See the Supporting Information, Figure S1 for the geometries of the transition states.

We tested our mechanochemical cascade using comb polymers, each containing either multiple DA adducts (**M2**) or STP moieties (**S2**, Scheme 1 and Scheme 2). A comb polymer contains a relatively short main chain with multiple three‐way branch points at which longer linear side chains are connected. In our polymers, the mechanophores were confined to the side‐chains in the proximity of the branch points. We chose comb polymers to maximize the efficiency of transducing external mechanical load to the force acting on individual mechanophores.[^45^, ^46^] Doing so is expected to reduce the minimum concentration of mechanophores needed to elicit a detectable stress response, which is critical for practical applications of such polymers.

**Scheme 2:**
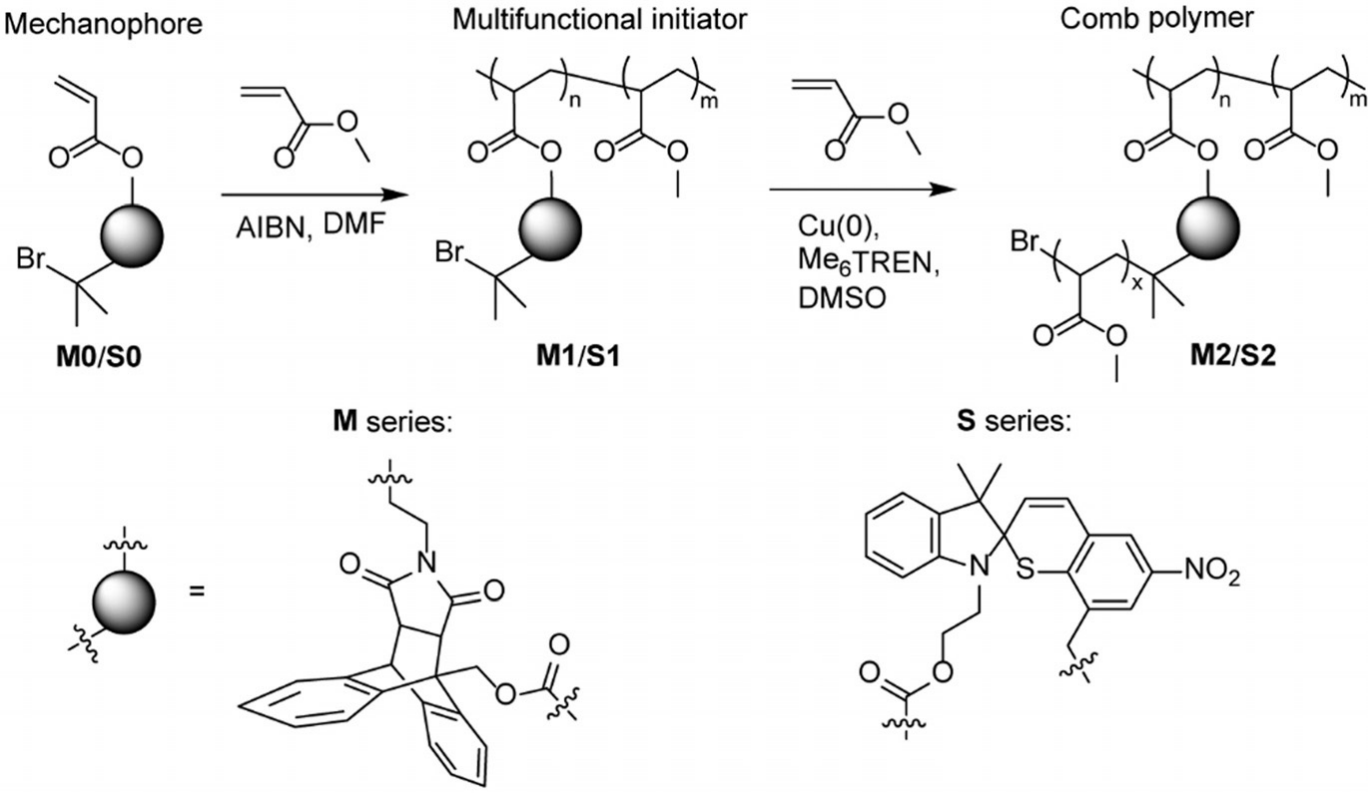
Syntheses of comb polymers **M2** and **S2**. AIBN: 2,2′‐azobis(2‐methylpropionitrile), DMF: N,N‐dimethylformamide, Me_6_TREN: tris[2‐(dimethylamino)ethyl]amine, DMSO: dimethylsulfoxide.

We prepared multifunctional initiators **M1** and **S1** by polymerizing mixtures of methyl acrylate and either **M0** (2:1 mol) or **S0** (10:1 mol) under standard conditions.^[47]^ The low fraction of **S0** in polymerization mixtures reflects our assumption that fairly demanding syntheses of spiropyrans necessitate minimizing their contents in polymers for practical applications, while maintaining an acceptable intensity of mechanochemical response. A combination of ^1^H NMR spectroscopy and size exclusion chromatography (SEC) suggests that an average chain of **M1** contained 18±3 DA adducts, branching and initiator moieties (*Ð*_M_=1.6; see the Supporting Information, Table S1 for further details); the equivalent number for **S1** was 15±2 (*Ð*_M_=1.7). Living radical polymerization of methyl acrylate yielded comb polymers **M2** and **S2** (Scheme 2 and Supporting Information) from multifunctional initiators **M1** and **S1**. The polymerization increased *M*_n_ of **M1** circa 100‐fold (**M2**: *M*_n_=1.1 MDa, *Ð*_M_=1.3; Supporting Information, Table S1) and that of **S1** circa 30‐fold (**S2**: *M*_n_=580 kDa, *Ð*_M_=1.7) or approximately proportionally to the estimated density of the initiator moieties in the two precursors, suggesting that the side chain in both **M2** and **S2** are of comparable size of about 50 kDa. A similar estimate of the side‐chain size is derived from the changes in the molecular mass distributions of these polymers during sonication (Supporting Information, Table S2 and below).

We confirmed mechanochemical generation of multiple anthracene‐terminated macromolecules from each chain of **M2** in sonicated dilute solutions of **M2** in THF under previously reported conditions (Figure 2 and Supporting Information).^[26]^ Aliquots of the sonicated solution were periodically withdrawn and analyzed by SEC, absorption, fluorescence, and ^1^H NMR spectroscopy. Several pieces of evidence unambiguously support mechanochemical cleavage of the DA adducts. First, SECs of sonicated solutions manifested a new peak at about 57 kDa, whose intensity relative to that of the reactant peak increased with the sonication time (Supporting Information, Figure S13). Second, sonicated solutions absorbed at 260–400 nm and fluoresced at 375–500 nm under 256 nm excitation (Supporting Information, Figure S14), with the intensities increasing during sonication. Both spectra were characteristic of anthracene.^[17]^ Third, ^1^H NMR spectra of sonicated **M2** contained new resonance signals at 6.58, and 8.05, 8.35, 8.52 ppm, consistent with those of maleimide and anthracene (Figure 2 b). Under the same conditions, sonication of the precursor to **M2**, **M1**, in which the DA adducts terminate very short side chains generated neither new ^1^H NMR resonances, absorbance nor fluorescence (Supporting Information, Figure S15). The difference supports mechanochemical rather than purely thermal mechanism^[39]^ of dissociation of the DA adducts of **M2**, which is precluded in **M1** whose adducts are not strained when the chain in stretched in an elongational flow.

**Figure 2:**
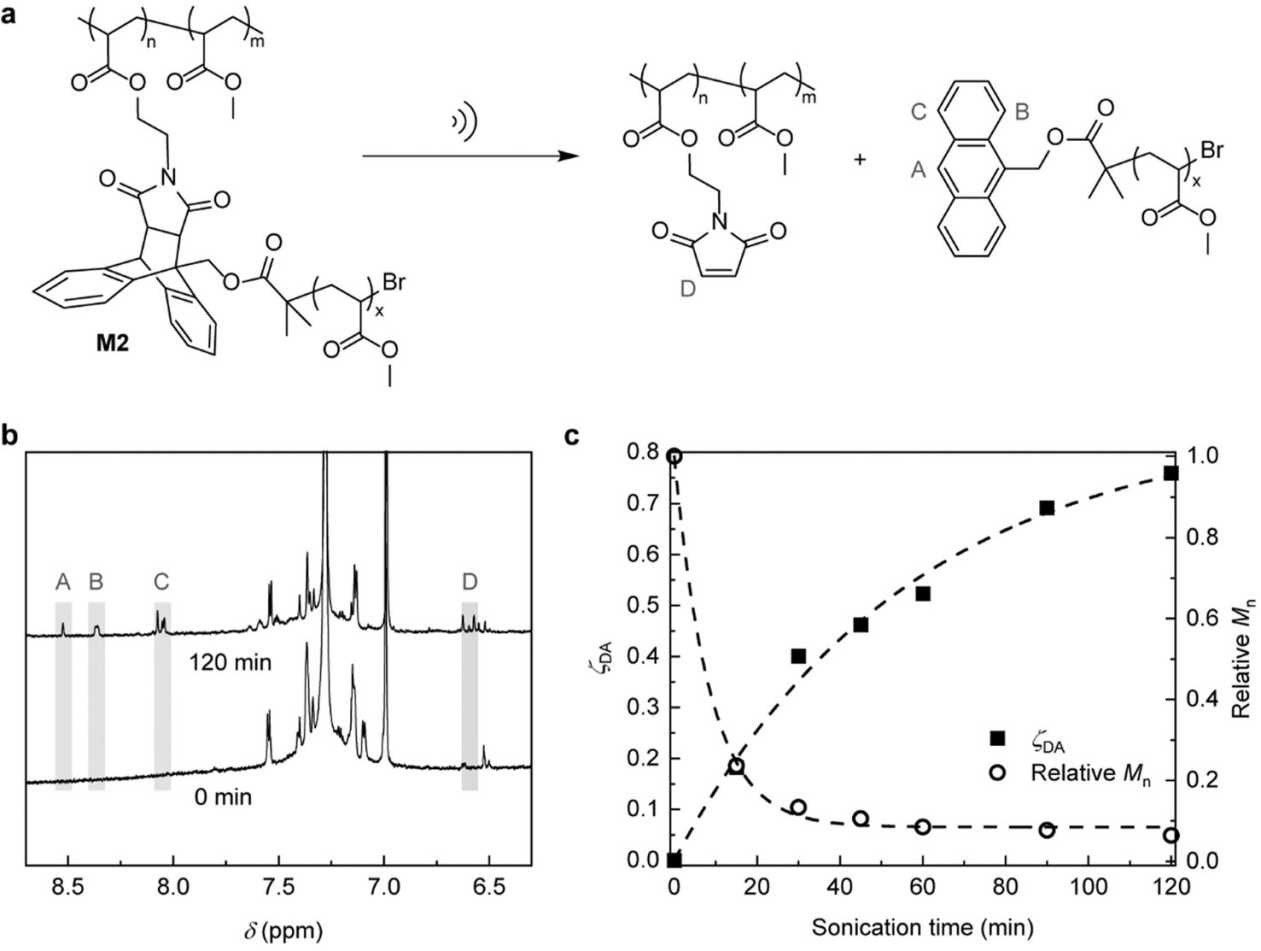
a) Mechanochemical retro‐DA reaction of comb polymer **M2** with anthracene‐maleimide moieties in solution. b) ^1^H NMR spectra of **M2** before and after 120 min sonication (5 mg mL^−1^, THF, 10 W cm^−2^, 0–5 °C, 1 s on and 1 s off), showing the products of the retro‐DA reaction as indicated by grey rectangles. c) The fraction of dissociated DA adducts, *ζ*_DA_, and the number‐average molar mass of sonicated solution of **M2**, relative to that of intact **M2**, as a function of sonication time. The dashed lines are guide for eyes.

The average molar mass of sonicated **M2** decreased during sonication (Figure 2 c; Supporting Information, Table S2) due to both gradual loss of side‐chains by dissociation of the DA adducts and non‐selective fragmentation of the backbone, which accompanies sonication of any polymer.^[39]^ The latter was evidenced by the appearance of a SEC peak at half the mass of intact **M2** (Supporting Information, Figure S13a), with negligible absorbance at >300 nm. To understand how both selective and non‐selective fragmentation of **M2** affects the kinetics of maleimide production, which controls the rate of bimolecular crosslinking, we analyzed the changes in *M*_n_, and the fraction of dissociated DA adducts, *ζ*_DA_, (Figure 2 c, Figure 3; Supporting Information, Figures S16–S21) during sonication. We used a kinetic scheme whereby a comb polymer containing *n* side chains (and hence *n* DA adducts), R_*n*_ (*n*>1), fragments either selectively to a chain with one fewer arms, R_*n*−1_, and a linear, anthracene terminated chain, P_An_, or non‐selectively to two comb macromolecules, each with half the original number of side chains (or, for odd *n*, into two macromolecules whose number of side chains differed by 1 and totaled *n*). The measured correlation between the number average molar mass and the fraction of dissociated DA adducts during sonication was reproduced accurately (solid line, Figure 3 a) by the simplest version of this Scheme in which the rate constant for fragmentation of R_*n*_ by mechanochemical dissociation of one of its *n* DA adducts was proportional to *n* (*n*>1), and non‐selective fragmentation contributes <7 % to the total reaction.

**Figure 3:**
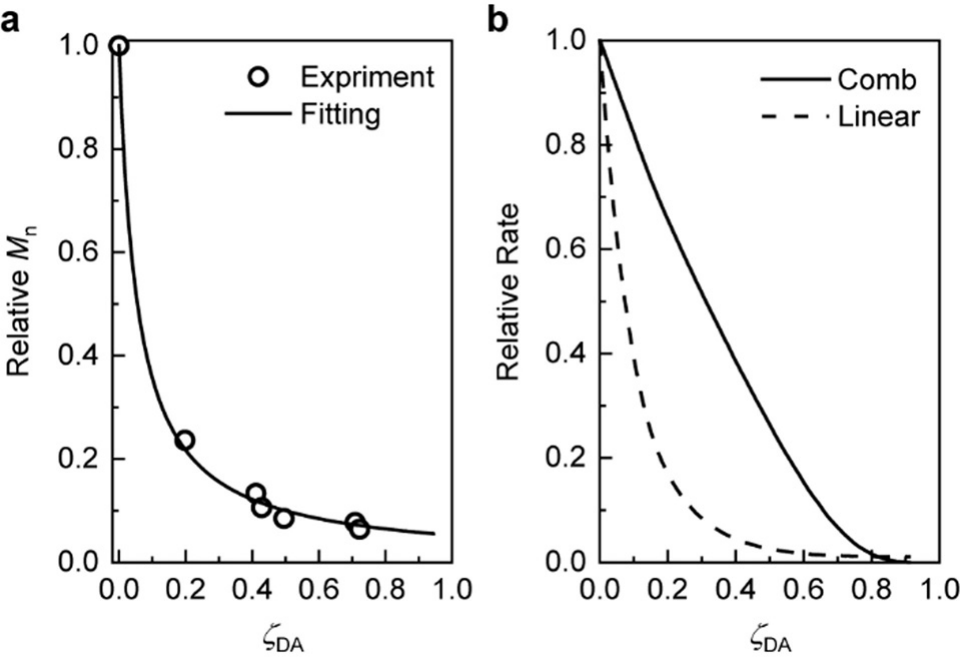
Modeling of mechanochemical kinetics of dissociation of the DA adduct in comb polymers. a) The measured correlation between the fraction of dissociated adducts, *ζ*_DA,_ and the average molar mass of the sonicated material, *M*_n_ (red dots) is reproduced by a simple model (black line). b) The relative rate of maleimide production by mechanochemical activation of a comb polymer (black line) is much less sensitive to the degree of conversion than that of a linear analog (red line).

Our modeling illustrates the advantage of using a comb polymer rather than a linear polymer for activation of scissile mechanophores whose products are used as reactants in a downstream reaction. The rate at which this reactant is generated by dissociation of a comb polymer decreases linearly with the fraction of the reacted moieties (solid line, Figure 3 b) and is largely insensitive to non‐selective fragmentation of the polymer. In contrast, this rate decreases exponentially with every fragmentation of a linear polymer (dash line, Figure 3 b), whether selective (that is, it generates the desired precursor for the subsequent downstream reaction) or non‐selective. The reason is that fragmentation of linear polymers either by dissociation of a scissile mechanophore or non‐selective homolysis of a backbone bond between mechanophores yields two fragments, each approximately half of length of the original chain. Because mechanochemical kinetics scales quadratically with the polymer contour length,^[39]^ activation of the remaining mechanophores in these fragments is considerably less efficient than in the original chain. In contrast, selective fragmentation of a multi‐mechanophore comb macromolecule produces another comb macromolecule, which is only marginally less sensitive to further mechanochemical activation because its spanning length remains intact. Moreover, non‐selective fragmentation of a comb polymer produces two new comb polymers whose combined reactivity matches that of the original polymer, according to our model.

Behavior of **S2** in sonicated solutions resembled that of its linear analogues,^[26]^ including reduction in the molar mass (Supporting Information, Figures S22, S23), rapid reaction with free N‐ethyl maleimide accompanied by a loss of the STP‐characteristic absorption (Supporting Information, Figure S24) and reversible photochromism (Supporting Information, Figure S25).

Sonication of a mixed solution of the two polymers (**M2** at 20 mg mL^−1^ and **S2** at 50 mg mL^−1^ initial concentrations) in DMF for 5 h yielded a suspension of a dark‐green material (Figure 4 b; Supporting Information, S26), which when isolated weighted 28 % of the total mass of the initially dissolved polymers. The material was insoluble in all solvents tested (Supporting Information, Figure S27), including those in which **M2** and **S2** dissolved readily even upon heating, suggesting extensive crosslinking (Supporting Information, Figure S28). The remaining solution manifested a fluorescence spectrum characteristic of anthracene (Figure 4 d; Supporting Information, Figure S29), the expected product of mechanochemical dissociation of the DA adducts of **M2**. The existence of the minimum concentration of **S2** below which crosslinking does not occur in sonicated solutions (Supporting Information, Figure S30) suggests a kinetic competition between a bimolecular addition of TMC to maleimide, the rate of which depends on the concentration of **S2**, and unimolecular isomerization of TMC to STP which is inert to reaction with maleimide.

**Figure 4:**
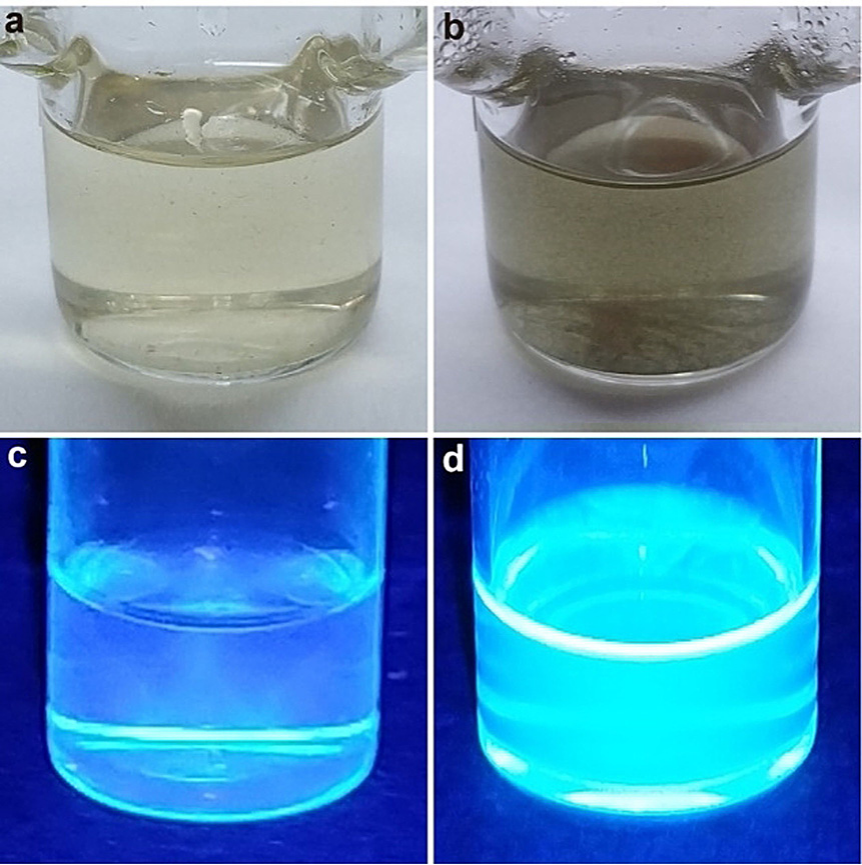
Mechanochemical cross‐linking of polymers **M2** and **S2** in sonicated THF solution. Optical (a) and fluorescence (c) images before sonication; Optical (b) and fluorescence (d) images after 5 h sonication.

A mixed solution of **M2** and **S2** at saturation concentrations that was not sonicated yielded no precipitate even under irradiation at 360 nm to photoisomerize STP to reactive TMC. Likewise, a film made of 1:1 mixture of **M2** and **S2** turned green upon UV irradiation, suggesting the generation of TMC, but remained fully soluble in DCM (Supporting Information, Figure S31). Both observations indicate the lack of crosslinking when **M2** is not subject to conditions necessary for mechanochemical dissociation of its DA adducts. In contrast, irradiating a mixture of sonicated **M2** (30 min sonication) and unsonicated **S2** at 365 nm for 30 min yielded insoluble materials (Supporting Information, Figure S32). A mixture of sonicated **M2** (30 min sonication) and unsonicated **S2** also became insoluble after 7 days at room temperature in the dark. The latter suggests that at room temperature the STP/TMC equilibrium maintains sufficient concentration of TMC to react with maleimide at an observable rate even in solids. Masking maleimide in the form of the DA adduct eliminates this spontaneous undesired crosslinking of the unloaded material.

The high cost of most reported mechanophores limits their potential practical applications and requires strategies to minimize mechanophore content without sacrificing the desired stress response. Although this problem has not been studied systematically, in all reported examples of crosslinking in sonicated solutions, the total concentration of mechanophores was on the order of about 100 mM.[^26^, ^27^, ^28^, ^29^] In contrast, sonicated solutions of **S2** and **M2** crosslink readily when the concentration of the respective moiety is as low as 1.6 mM and 0.35 mM, or about two orders of magnitude lower than previous examples. We assume that the primary reason is how sensitive the kinetics of mechanophore activation is to chain fragmentation in linear and comb polymers as discussed above.

Identifying strategies to enable simultaneous control over mechanochemical reactivity and the threshold force at which it occurs at practical rate remains a major unresolved problem in contemporary polymer mechanochemistry. The task is particularly challenging for materials that display multiple productive responses to load, because it requires integration of several mechanochemical reactions while avoiding cross‐reactivity. Above we described an intermolecular reaction cascade to increase by about 1 nN the threshold force that triggers crosslinking of a mechanochromic material containing spirothiopyran and Diels–Alder adducts of anthracene and maleimide. The onset of reversible green mechanochromism is determined by the mechanochemical kinetics of isomerization of spirothiopyran to thiomerocyanine, with half‐life of about 1 min at about 1 nN. The dye is reactive towards activated C=C double bonds, crosslinking spontaneously in their presence. Masking these bonds as DA adducts allows the onset of crosslinking to be governed by the dissociation kinetics of the DA adduct instead of the more labile STP‐to‐TMC isomerization. As a result, this cascade provides a means of limiting the occurrence of irreversible crosslinking to overstressed volumes of the loaded material that are at the highest risk of failure. The diverse reactivity of maleimide, including as a Michael's receptor and as a dienophile, and the relatively simple means of adjusting the dissociation kinetics of its DA adducts by substitution^[5]^ suggests that the approach described here is suitable for controlling a broad range of reaction sequences triggered by mechanical load.

## Conflict of interest

The authors declare no conflict of interest.

## Supporting information

As a service to our authors and readers, this journal provides supporting information supplied by the authors. Such materials are peer reviewed and may be re‐organized for online delivery, but are not copy‐edited or typeset. Technical support issues arising from supporting information (other than missing files) should be addressed to the authors.

Supplementary
