# Supplementary material for: A Mechanochemical Reaction Cascade for Controlling Load‐Strengthening of a Mechanochromic Polymer
Source: Angew Chem Int Ed Engl. 2020 Sep 25;59(49):21980–5. doi: 10.1002/anie.202010043 (PMC7756483; doi:10.1002/anie.202010043)
Supplement: Supplementary file 1 — Supplementary [file ANIE-59-21980-s001.pdf]

Angewandte Chemie
Eine Zeitschrift der Gesellschaft Deutscher Chemiker

Supporting Information

A Mechanochemical Reaction Cascade for Controlling Load-Strengthening of a Mechanochromic Polymer

Yifei Pan[+], Huan Zhang[+], Piaoxue Xu, Yancong Tian, Chenxu Wang, Shishuai Xiang, Roman Boulatov, and Wengui Weng**

anie_202010043_sm_miscellaneous_information.pdf

I. Quantum-chemical calculations of activation enthalpies and kinetic modelling

All calculations were performed with the Gaussian09.E01[1] software package. The Berny algorithm was used to locate stationary points. Very tight convergence criteria and ultrafine integration grids were used in all optimizations. Thermodynamic corrections to electronic energies of individual conformers were calculated statistical-mechanically in the harmonic oscillator/rigid rotor/ideal gas approximations, as $3RT + ZPE + U_{vib} - TS$, where ZPE is the zero-point energy, U_{vib} is the vibrational component of the internal energy and S is the total entropy. Vibrational frequencies below 500 cm^{-1} were replaced with 500 cm^{-1} as previously recommended[2], to avoid the artifactually large contribution of such low-frequency modes to vibrational entropy. The calculations of analytical frequencies on converged constrained molecules is valid because the molecule plus its infinitely-compliant constraining potential is a stationary point.[3-4] The enthalpies of ensembles were calculated as $G_{\min} - RT\ln\sum g_i e^{-\Delta G_i/RT}$, where $G_{\min}$ is the free energy of the conformational minimum, ΔG_i is the excess free energy of conformer i relative to this minimum, and g_i is its degeneracy. The energy barriers separating individual strain-free conformers were <4 kcal/mol, justifying the use of Boltzmann statistics in calculating properties of ground and transition states and energies of activation. Ensemble averaging was done as $\langle\alpha\rangle = \frac{\sum \alpha_i g_i e^{-\Delta G_i \beta}}{\sum g_i e^{-\Delta G_i \beta}}$, where α is the quantity of interest (e.g., end-to-end distance) and the remaining terms are defined above.

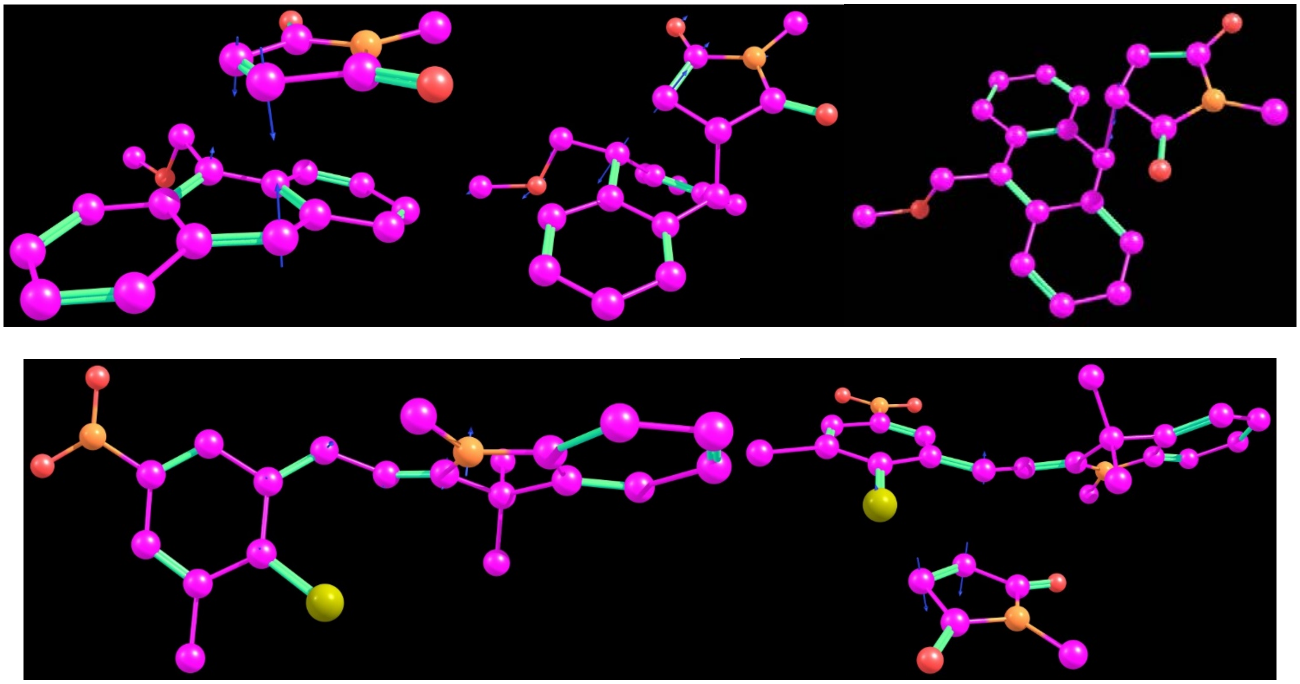

Figure S1. Converged geometries of the transition states (from left to right): concerted dissociation of the DA adduct at 0 pN; 1st and 2nd open-shell transition states for step-wise dissociation of the DA adduct at 1 nN; isomerization of STP to TMC; concerted addition of TMC to maleimide. The reactive motions are illustrated by blue arrows. All geometries are at CAM-B3LYP/6-311+G(d) in CPCM=THF (UHF for the open-shell structures).

The converged wavefunctions were stable as determined by outcome of the testing with the “stable” key word in Gaussian. All converged conformers of the reactant or intermediate states had 0 imaginary frequencies and all converged conformers of the transition states had a single imaginary frequency with the nuclear motion consistent with dissociation/rotation as appropriate. Unconstrained conformational ensembles of the DA adduct (6 conformers), including the transition states and an open shell intermediate, were built systematically as previously described.[5] The transition state conformers of the DA dissociation and of STP isomerization and TMC addition to maleimide were first optimized at B3LYP/6-31G(d) with constraints of the scissile or forming bonds (and for open-shell transition states, of the $_{Me}C \cdots C_{Me}$ distance defining the pulling axis and the UHF formalism), followed by analytical frequency calculations and optimizations at CAM-B3LYP/6-311+G(d) (with UHF formalism for the open-shell transition states). Force-dependent electronic energies, thermodynamic corrections and $_{Me}C \cdots C_{Me}$ distances of individual conformers and the free energies and distances of the conformational ensembles were calculated following the described procedures.[6]

Intrinsic reaction path calculations were performed from the TS for concerted dissociation at 0 pN and on both open-shell TSs of the step-wise mechanism (at 1 nN) of the DA adduct and from the TSs for STP isomerization and TMC additions. The final geometries of all IRC calculations optimized to the expected minima.

Kinetic modelling was performed in Matlab by numerical solution of the corresponding differential rate laws with ode45: $\frac{dR_\mathrm{m}}{d\left(\frac{t}{k}\right)} = -\left(m + \frac{k_\mathrm{ns}}{k}\right) R_\mathrm{m}$, $\frac{dR_\mathrm{n}}{d\left(\frac{t}{k}\right)} = (n+1)R_\mathrm{n+1} + \frac{k_\mathrm{ns}}{k}(2R_\mathrm{2n} + R_\mathrm{2n+1} + R_\mathrm{2n-1}) - \left(n + \frac{k_\mathrm{ns}}{K}\right) R_\mathrm{n}$ ($m<n<1$, the 2nd term only applies for $n \leq$floor(m/2)) and $\frac{dP_\mathrm{An}}{d\left(\frac{t}{k}\right)} = \sum_{i=2}^{m} iR_\mathrm{i}$, where k and k_ns are rate constants for chain fragmentation by dissociation of the DA adduct and homolysis of any other backbone bond, R_n and P_{An} are chain fractions of a comb polymer containing n DA adducts and of linear, anthracene terminated polymer, respectively and m is the maximum number of side chains in the largest comb polymer. The M_n and degree of conversion, ζ_{DA} were calculated as: $M_n = M_\mathrm{s} \sum_{i=1}^{m} iR_\mathrm{i} + M_\mathrm{b} / \left(2 - \frac{P_\mathrm{An}}{\sum_{i=1}^{m} iR_\mathrm{i}}\right)$ and $\varsigma_\mathrm{DA} = m/P_\mathrm{An}$, where M_s and M_b are molar masses of the average side-chain and the backbone. The fitting used the lsqnonlin function of Matlab with M_s, M_b, k, and k_ns being the fitting parameters (m was varied systematically from 22 to 13). The converged LSF with the minimum resnorm corresponded to M_s = 56±4 kDa M_b = 8.2±0.9 kDa; m = 18; k= 1; k_ns = 0.07±0.03 (the rate constants are unitless because simulation was performed in normalized time, t = 1/k). The errors were calculated from the Jacobian returned by lsqnonlin. In calculating the relative rate of release of maleimide from a linear polymer, quadratic scaling of the fragmentation rate of chain size was assumed.

II. Experimental details

Materials

Maleic anhydride, ethanolamine, 9-anthracene methanol, 4-dimethyl-aminopyridine, 2-bromo isobutyryl bromide, methyl acrylate, 2,2'-azobis(2-methylpropionitrile) (AIBN) , tris[2-(dimethylamino)ethyl]amine (Me_6TREN), acryloyl

chloride 5-nitrosalicylaldehyde, methacrylic acid, *N,N*-dimethylthiocarbamoyl chloride, *N*-ethylmaleimide, 2-Iodoethanol, 2,3,3-trimethylindoleniune were purchased from Energy. Tetrahydrofuran (THF) was dried with Na before use. Dichloromethane (DCM), N,N-dimethylformamide (DMF), dimethylsulfoxide (DMSO), toluene were distilled over CaH_2 under nitrogen. All the other reagents were purchased from Sinopharm and used without further purification.

NMR

^{1}H NMR and ^{13}C NMR spectra were recorded in $CDCl_3$ (δ= 7.26 (^{1}H) and 77.16 (^{13}C)) and referenced to the residual solvent signals on a 500 MHz Brucker AvanceII spectrometer at 25 °C. All chemical shifts were given in ppm (δ) as singlet (s), doublet (d), triplet (t), quartet (q), multiplet (m), or broad (br).

SEC

Size exclusion chromatography (SEC) data were calibrated on two columns in series (7.8 × 300 mm, 2 GMHHRM17932 and 1 GMHHRH17360) with tetrahydrofuran (THF, HPLC grade) as eluent at 40 °C with a LC-20AD pump. The facility was equipped with two detectors (RID-10A refractive index detector; SPD-20A UV detector) and the molecular weight was calibrated against polystyrene standards. See the subsequent section for the description of how molar masses of the polymers were estimated.

FTIR

Attenuated total reflection-Fourier transform infrared spectroscopy (ATR-FTIR) data were collected using a Nicolet Avatar 360 with an Omni-Sampler ATR accessory (Ge crystal, single-bounce beam path, 45° incident angle, 32 scans, 4 cm^{-1} resolution). An advanced ATR correction was applied to all spectra.

Small Molecule Synthesis

Synthesis of anthracene/maleimide initiator (M0)

Maleic anhydride (5.0 g, 51 mmol) was dissolved in diethyl ether (25 mL) and furan (18.5 mL, 255 mmol) was slowly added while stirring. The reaction mixture was stirred for 48 h at room temperature. Then the solvent was removed under reduced pressure to provide the product **1** as a colorless powder (7.9 g, 48 mmol, 94.0% yield).^{1}H NMR ($CDCl_3$, 500 MHz): δ(ppm) = 6.60 (s, 2H), 5.48 (s, 2H), 3.19 (s, 2H).

The Diels-Alder adduct **1** (5.0 g, 30 mmol) was dissolved in methanol (125 mL). The solution was purged with N_2 for 10 min while immersed in an ice bath. Ethanolamine (2 mL, 33 mmol, 1.1 eq.) was added as well as triethylamine (4.2 mL, 30 mmol). The temperature was increased to 67 °C and the reaction mixture was stirred. After 13 h, 10% additional ethanolamine (0.2 mL, 3 mmol) was added and the temperature was increased to 70 °C over 2 h. The reaction mixture was cooled to room temperature and white crystals precipitated. They were washed with isopropyl

alcohol to obtain compound **2** (4.0 g, 19 mmol, 63.0% yield). ^{1}H NMR ($CDCl_3$, 500 MHz): δ(ppm) = 6.54 (s, 2H), 5.30 (s, 2H), 3.78 - 3.71 (dt, *J* = 42.3 Hz, 4H), 2.91 (s, 2H), 1.27 (s, 1H).

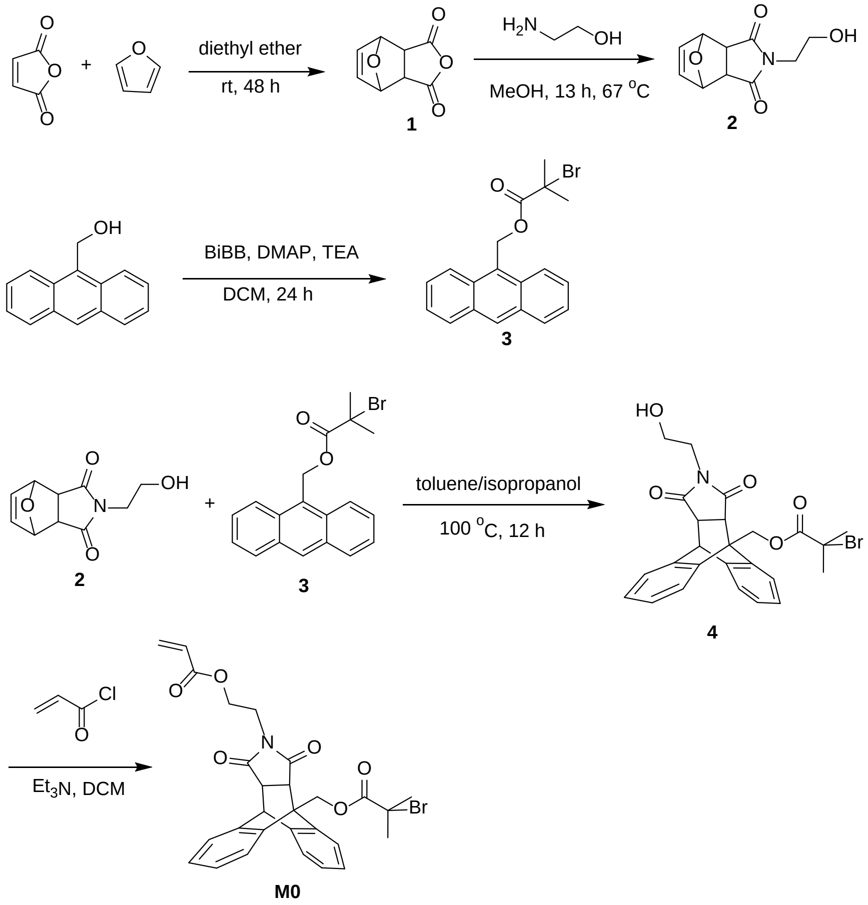

A solution of 9-anthracene methanol (10.0 g, 48 mmol) and 4-dimethyl-aminopyridine (586 mg, 4.8 mmol) in dichloromethane (DCM, 240 mL) was prepared. Triethylamine (Et_3N, 8.4 mL, 60 mmol) was added and the reaction mixture was cooled to 0 °C, 2-bromo isobutyryl bromide (BiBB, 6 mL, 60 mmol) was added dropwise over the course of 5 min. The reaction mixture was stirred for 1 h at 0 °C and then for 24 h at room temperature. The resultant solution was washed with water, a saturated aqueous solution of $NaHCO_3$, and then again with water. Traces of water were removed by drying over $MgSO_4$. Solvent removal under reduced pressure yielded a yellow oil. Purification by column chromatography (silica, cyclohexane/ethyl acetate = 9/1 (v/v)) provided a light yellow solid **3** (15.0 g, 42 mmol, 87.5% yield). ^{1}H NMR ($CDCl_3$, 500 MHz): δ(ppm) = 8.55 (s, 1H), 8.38 - 8.36 (d, *J* = 8.74 Hz, 2H), 8.07 - 8.05 (d, *J* = 8.52 Hz, 2H), 7.61 - 7.52 (dt, *J* = 42.7 Hz, 4H), 6.25 (s, 2H), 1.90(s, 6H).

Compound **2** (1.5 g, 7 mmol) and compound **3** (2.5 g, 7 mmol) were added into 50 mL flask and charged with 15 mL toluene and 10 mL isopropanol. The solution was refluxed for 12 h and cooled down before removal of solvents under vacuum. Residual raw product was purified by chromatography (silica, hexane/ethyl acetate = 2/1 (v/v)) at first to remove the low polarity **2** and then (silica, hexane/ethyl acetate= 1/2 (v/v)) to yield the desired product **4** as

a white solid in 83% yield. ^1H NMR ($CDCl_3$, 500 MHz): δ(ppm) = 7.42 (m, 3H), 7.28 (m, 5H), 5.66 (s,1H), 5.52 (s, 1H), 4.85 (s, 1H), 3.36 (s, 4H), 3.12 (s, 2H), 2.01 (s, 6H).

Compound **4** (2.5 g, 5 mmol) was added into a 150 mL flask and charged with 50 mL dichloromethane (DCM) followed by addition of triethylamine (Et_3N) (1.4mL, 10 mmol). The flask was immersed in ice bath and acryloyl chloride (0.6 mL, 7.5 mmol) was added dropwise using additional funnel. The solution was stirred for additional 1 h and raised to room temperature for 12 h. The resultant solution was washed using DI water, $NaHCO_3$, DI water to remove acid chloride and water soluble impurity. The solution was dried using $MgSO_4$ and solvents were removed under vacuum. The crude product was purified by chromatography (silica, hexane/ethyl acetate = 2/1 (v/v)) to yield the desired product **M0** as a white solid in 73% yield. ^1H NMR ($CDCl_3$, 500 MHz): δ(ppm) = 7.44 - 7.16 (m, 8H), 6.41 - 6.37 (d, *J* = 17.25 Hz, 1H), 6.05 (m, 1H), 5.90 - 5.88 (d, *J* = 10.56 Hz, 1H), 5.66 (s, 1H), 5.48 (s, 1H), 4.82 (s, 1H), 3.64 -3.54 (m, 2H), 3.46 - 3.43 (t, *J* = 11.45 Hz, 2H), 3.36 (s, 2H), 2.02 - 2.00 (d, *J* =10.68 Hz, 6H).

Synthesis of spirothiopyran (STP) diol (8)

3-formyl-2-mercapto-5-nitrobenzyl methacrylate (**5**) and 9,9,9a-trimethyl-2,3,9,9a-tetrahydrooxazolo[3,2 -a]indole (**6**) were synthesized according to the previous literatures.[7]

(1-(2-hydroxyethyl)-3,3-dimethyl-6'-nitrospiro[indoline-2,2'-thiochromen]-8'-yl)methyl methacrylate (**7**): **5** (562 mg, 2 mmol) and **6** (487 mg, 2.4 mmol) were refluxed in ethanol for 12 h. The solvent was then removed under vacuum. The product was further purified by flash column chromatography with ethyl acetate/hexane (1/2) as the eluent. Yellow oil **7** was obtained (475 mg, 1.02 mmol, 51.0% yield).^1H NMR ($CDCl_3$, 500 MHz): δ(ppm) = 8.09 - 8.08 (d, *J* = 2.35 Hz, 1H), 8.00 - 7.99 (s, *J* = 2.4 Hz, 1H), 7.18 - 7.15 (t, *J* = 7.73 Hz, 1H), 7.09 - 7.07 (d, *J* = 7.25 Hz, 1H), 7.92 - 6.89 (m, 2H), 6.61 - 6.59 (d, *J* = 7.85 Hz, 2H), 6.17 (s, 1H), 6.10 - 6.07 (d, *J* = 11 Hz, 1H), 5.63 - 5.62 (m, 1H), 5.21 - 5.18(d, *J* = 13.8 Hz, 1H), 5.11 - 5.08 (d, *J* = 13.75 Hz, 1H), 3.86 - 3.77 (m, 2H), 3.55 - 3.49 (m, 1H), 3.25 - 3.20(m, 1H), 1.96 (s, 3H), 1.37 (s, 3H), 1.25 (s, 3H).

2-(8'-(hydroxymethyl)-3,3-dimethyl-6'-nitrospiro[indoline-2,2'-thiochromen]-1-yl)ethan-1-ol (**8**): To a solution of compound **7** (466 mg, 1 mmol) in 30 mL CH_3OH/CH_2Cl_2 (1/1) mixture K_2CO_3 (1.38 g, 10 mmol) was added and then the resulting solution was stirred for 5 h. HCl (aqueous, 1 M) was added into the reaction mixture, and the product was extracted with ethyl acetate. The extract was washed with saturated NaCl (aqueous) and dried over anhydrous sodium sulfate. Then the solvent was removed under vacuum and the crude product was purified with column chromatography (ethyl acetate/hexane = 1 : 2) to give (359 mg, 1.06 mmol, 90% yield) spirothiopyran (STP) diol **8** as a yellow solid.^1H NMR ($CDCl_3$, 500 MHz): δ(ppm) = 8.20 - 8.19 (d, *J* = 2.40 Hz, 1H), 7.97 - 7.96 (d, *J* = 2.45 Hz, 1H), 7.18 - 7.15 (t, *J* = 7.68 Hz, 1H), 7.10 - 7.08 (d, *J* = 7.20 Hz, 1H), 6.91 - 6.88 (m, 2H), 6.60 - 6.58 (d, *J* = 7.8 Hz, 1H), 4.68 - 4.65 (m, 2H), 3.83 - 3.87 (m, 2H), 3.52 - 3.50 (m, 1H), 3.22 - 3.18 (m, 1H), 2.11 (s, 1H), 1.75 (s, 1H), 1.36 (s, 3H), 1.26 (s, 3H).^{13}C NMR ($CDCl_3$, 500 MHz): δ(ppm) = 147.11, 144.51, 140.25, 138.70, 129.25, 127.93, 124.82, 122.45, 122.10, 120.86, 120.21, 107.14, 89.64, 61.56, 60.78, 53.04, 48.06, 24.18, 22.29.

Synthesis of spirothiopyran initiator (S0)

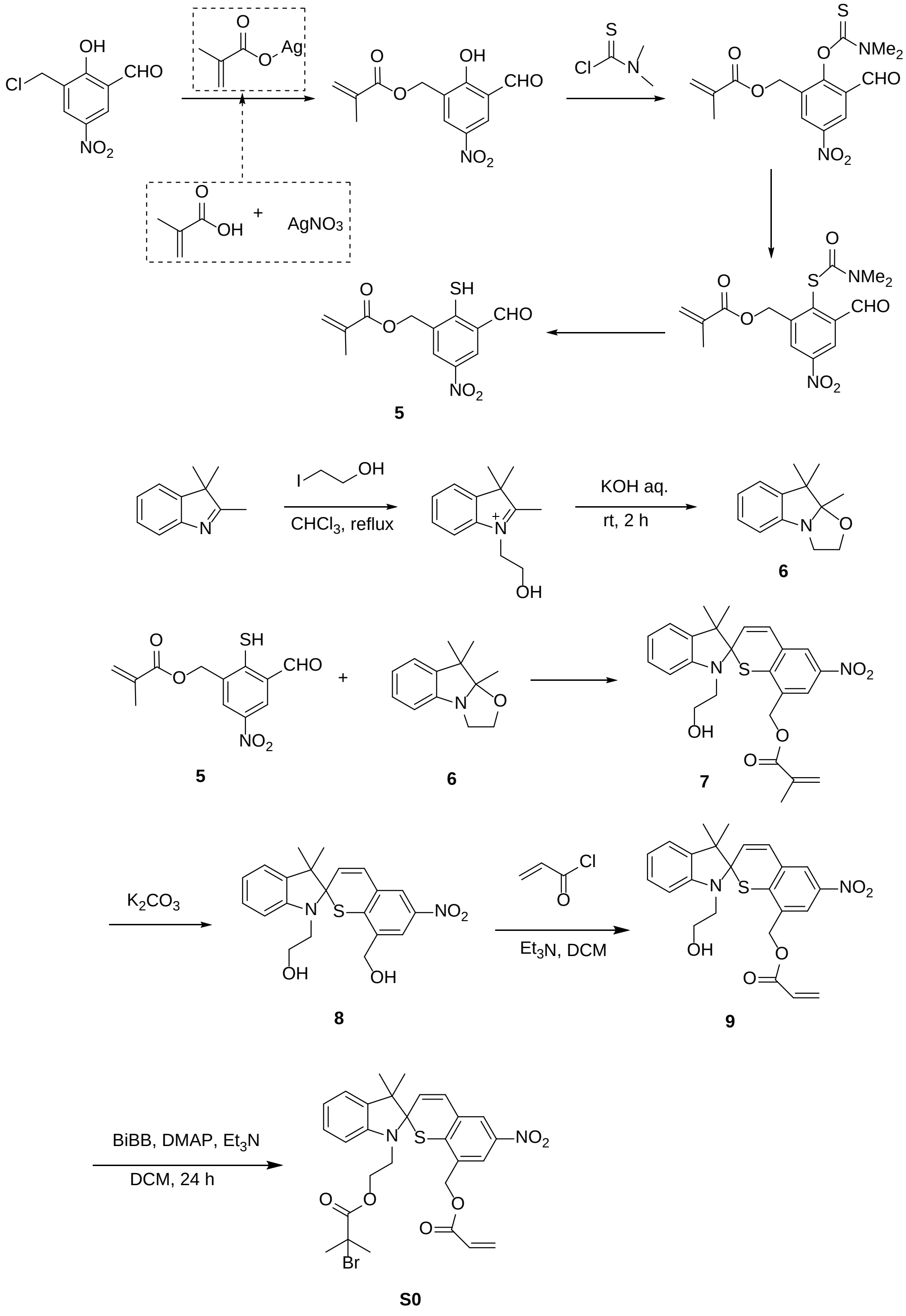

Compound **8** (0.21 g, 0.53 mmol) was added into a 10 mL flask and charged with 5 mL dichloromethane (DCM) followed by addition of triethylamine (Et_3N) (73 µL, 0.53 mmol). The flask was immersed in ice bath and acryloyl chloride (42 µL, 0.53 mmol) was added dropwise using additional funnel. The solution was stirred for additional 1 h and raised to room temperature for 12 h. The resultant solution was washed using DI water, $NaHCO_3$, DI water to

remove acid chloride and water-soluble impurity. The solution was dried using $MgSO_4$ and solvents were removed under vacuum. The crude produce was purified by chromatography (silica, hexane/ethyl acetate = (2/1)) to yield the desired product **9** as a yellow solid in 68% yield. ^{1}H NMR ($CDCl_3$, 500 MHz): δ(ppm) = 8.08 - 7.99 (dd, *J* = 43.3 Hz, 2H), 7.21 - 7.08 (m, 2H), 6.92 - 6.89 (m, 2H), 6.66 - 6.65 (d , *J* = 7.83 Hz, 1H), 6.45 - 6.41 (dd, *J* = 18.8 Hz, 1H), 6.17 - 6.12 (m, 1H), 6.07 - 6.05 (d, *J* = 11 Hz, 1H), 5.89 - 5.87 (dd, *J* = 11.9 Hz, 1H), 5.15 - 5.00 (dd, *J* = 74.1 Hz, 2H), 4.44 - 4.31 (m, 2H), 3.72 - 3.34 (m, 2H), 1.37 - 1.21(d, *J* = 76.7 Hz, 6H).

A solution of **9** (50 mg, 0.11 mmol) and 4-dimethyl-aminopyridine (1.3 mg, 0.011 mmol) in dichloromethane (2 mL) was prepared. Triethylamine (Et_3N) (20 µL, 0.14 mmol) was added and the reaction mixture was cooled to 0 °C, 2-bromo isobutyryl bromide (18 µL, 0.14 mmol) was added. The reaction mixture was stirred for 1 h at 0 °C and then for 24 h at room temperature. The resultant solution was washed with water, a sat. aq. soln. of $NaHCO_3$, and then again with water. Traces of water were removed by drying over $MgSO_4$. Solvent removal under reduced pressure yielded a yellow oil. Purification by column chromatography (silica, cyclohexane/ethyl acetate = 3/1 (v/v)) provided a yellow solid **S0** (52 mg, 0.086 mmol, 79% yield). ^{1}H NMR ($CDCl_3$, 500 MHz): δ(ppm) = 8.08 - 7.99 (dd, *J* = 43.3 Hz, 2H), 7.21 - 7.08 (m, 2H), 6.92 - 6.89 (m, 2H), 6.66 - 6.65 (d , *J* = 7.83 Hz, 1H), 6.45 - 6.41 (dd, *J* = 18.8 Hz, 1H), 6.17 - 6.12 (m, 1H), 6.07 - 6.05 (d, *J* = 11 Hz, 1H), 5.89 - 5.87 (dd, *J* = 11.9 Hz, 1H), 5.15 - 5.00 (dd, *J* = 74.1 Hz, 2H), 4.44 - 4.31 (m, 2H), 3.72 - 3.34 (m, 2H), 2.97 - 2.94 (d, *J* = 14.7 Hz, 6H), 1.37 - 1.21(d, *J* = 76.7 Hz, 6H).

Polymer Synthesis

Synthesis of anthracene/maleimide containing polymers M1 and M2

Compound **M0** (1.66 g, 3 mmol), methyl acrylate (0.6 g, 7 mmol), 2,2'-Azobis(2-methylpropionitrile) (AIBN, 16.4 mg, 0.1 mmol), and 6 mL DMF were added to a 15 mL Schlenk flask. Three freeze-pump-thaw cycles were carried out to remove oxygen. Then, the Schlenk flask was allowed to stir under argon at 70 °C for 12 h. After cooling to room temperature, the polymer solution was added dropwise to stirred, ice-cold methanol. Methanol was decanted and the polymer solid redissolved in DCM. After repeating the precipitation process 3 times, the obtained polymer solid was dried under vacuum to afford a white solid **M1** (1.97 g, 87% yield). ^{1}H NMR of the product revealed only negligible incorporation of methyl acrylate in **M1**, which probably explains the modest degree of polymerization of **M1**. MALDI-TOF analysis of **M1** revealed the presence of oligomers of **M0** of up to 18-mer, as evidenced by multiple peaks separated by 557 amu, with the mean mass of ~5.1 kDa. MALDI analysis of polymer samples was previously noted[8] to yield lower mean molar masses compared to SEC. See Table S1 above for the results SEC characterization.

Methyl acrylate (3.4 mL, 37.79 mmol), Me_6TREN (1.3 µL, 5.2 µmol), Cu(0) (10mg), the initiator **M1** (20 mg, 2.6 µmol), DMSO (3.4 mL) were added in a Schlenk flask. Three freeze-pump-thaw cycles were carried out to remove oxygen. Then, the Schlenk flask was allowed to stir under argon at room temperature for 5 h. Then, the polymer solution was added dropwise to stirred, ice-cold methanol. Methanol was decanted and the polymer solid

redissolved in DCM. After repeating the precipitation process 3 times, the obtained polymer solid was dried under vacuum to afford a white transparent gel **M2**. See Table S1 above for the results SEC characterization.

Synthesis of STP-containing polymers S1 and S2

Compound **S0** (26 mg, 0.043 mmol), methyl acrylate (34 mg, 0.39 mmol), 2,2'-Azobis(2-methylpropionitrile) (AIBN, 0.7 mg, 0.0043 mmol), and 0.5 mL DMF were added to a 5 mL Schlenk flask. Three freeze-pump-thaw cycles were carried out to remove oxygen. Then, the Schlenk flask was allowed to stir under argon at 70 °C for 12 h. After cooling to room temperature, the polymer solution was added dropwise to stirred, ice-cold methanol. Methanol was decanted and the polymer solid redissolved in DCM. After repeating the precipitation process 3 times, the obtained polymer solid was dried under vacuum to afford a yellow solid **S1** (35 mg, 58% yield). See Table S1 above for the results SEC characterization. The complex and crowded ^{1}H NMR spectrum of STP precludes quantitation of the number of STP units per average chain of **S1** from ^{1}H NMR analysis of **S1**; this number was derived by assuming the ratio of the two repeat units in **S1** equal that in monomer feed.

Methyl acrylate (0.91 mL, 10.2 mmol), Me_6TREN (0.1 µL, 0.43 µmol), Cu(0) (1 mg), the initiator **S1** (6 mg, 0.72 µmol), DMSO (0.91 mL) were added in a Schlenk flask. Three freeze-pump-thaw cycles were carried out to remove oxygen. Then, the Schlenk flask was allowed to stir under argon at room temperature for 5 h. Then, the polymer solution was added dropwise to stirred, ice-cold methanol. Methanol was decanted and the polymer solid redissolved in DCM. After repeating the precipitation process 3 times, the obtained polymer solid was dried under vacuum to afford a white transparent gel **S2**. See table S1 above for the results SEC characterization.

III. NMR Spectra

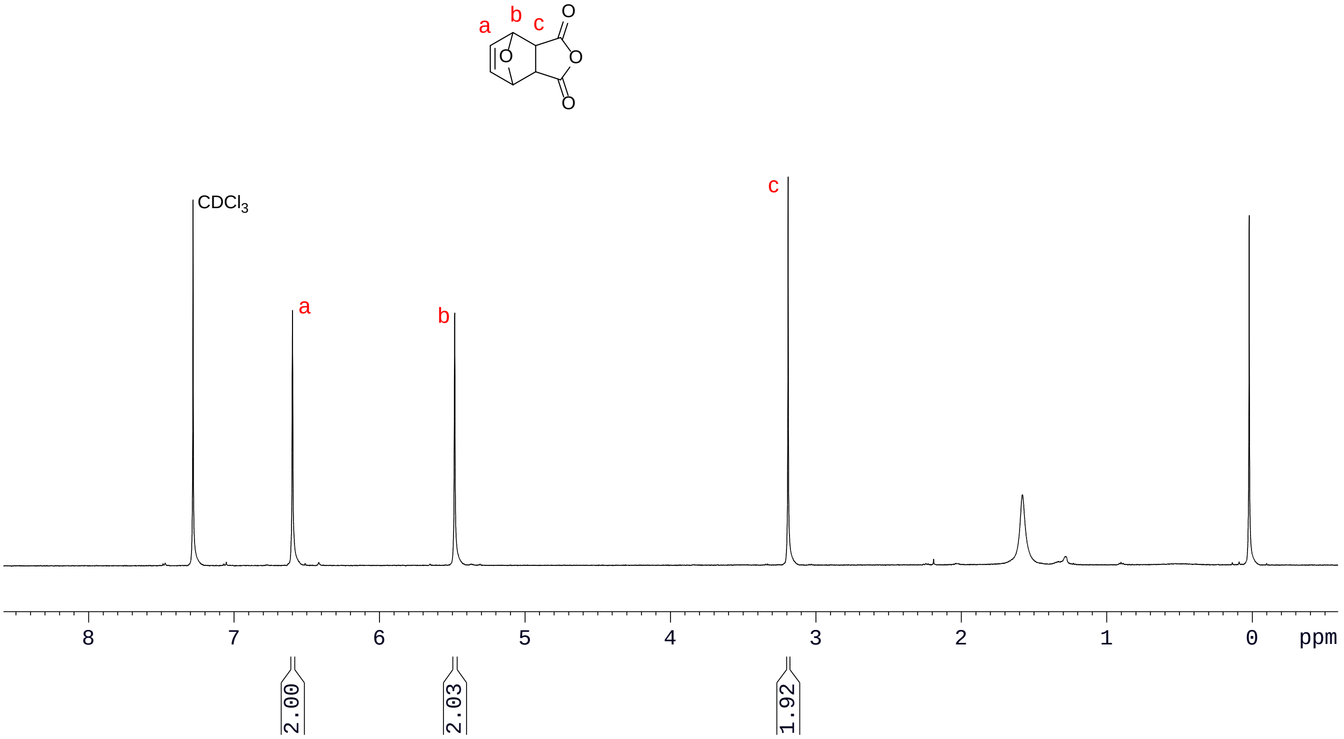

Figure S2. ^{1}H NMR (500 M) spectrum of compound **1** ($CDCl_3$, 7.26 ppm).

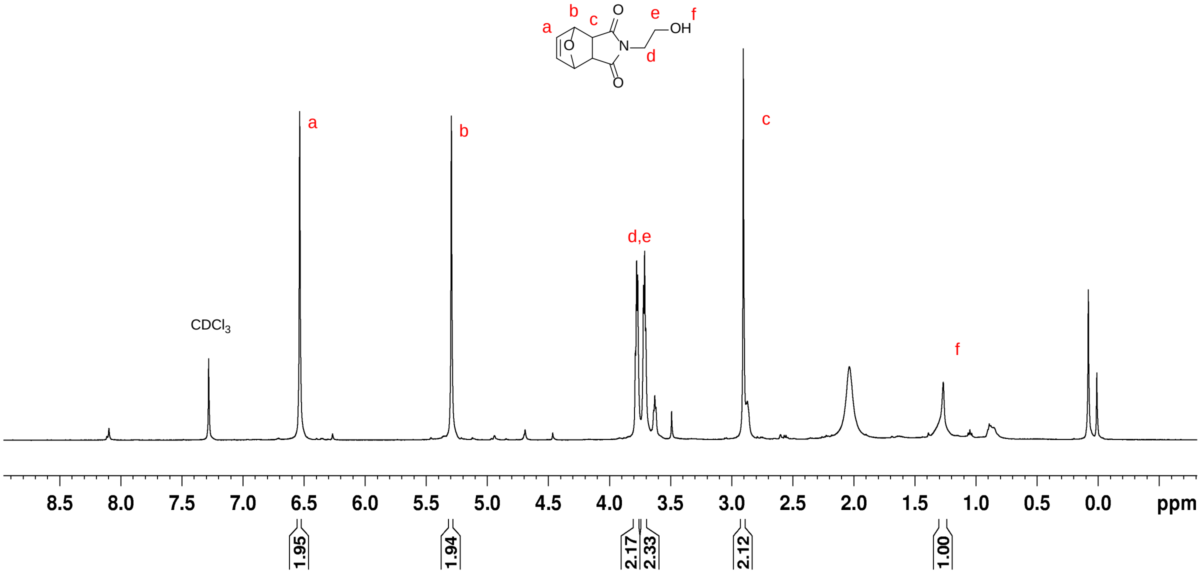

Figure S3. ^{1}H NMR (500 M) spectrum of compound **2** ($CDCl_3$, 7.26 ppm).

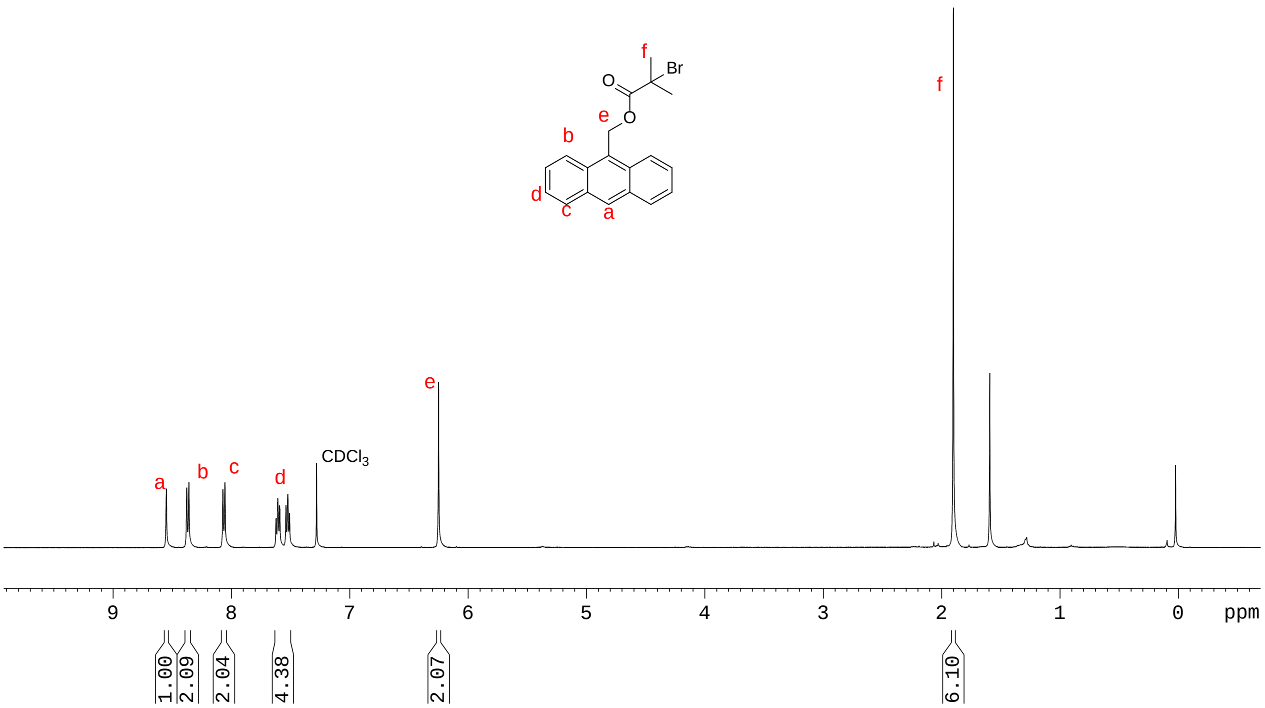

Figure S4. ^{1}H NMR (500 M) spectrum of compound **3** ($CDCl_3$, 7.26 ppm).

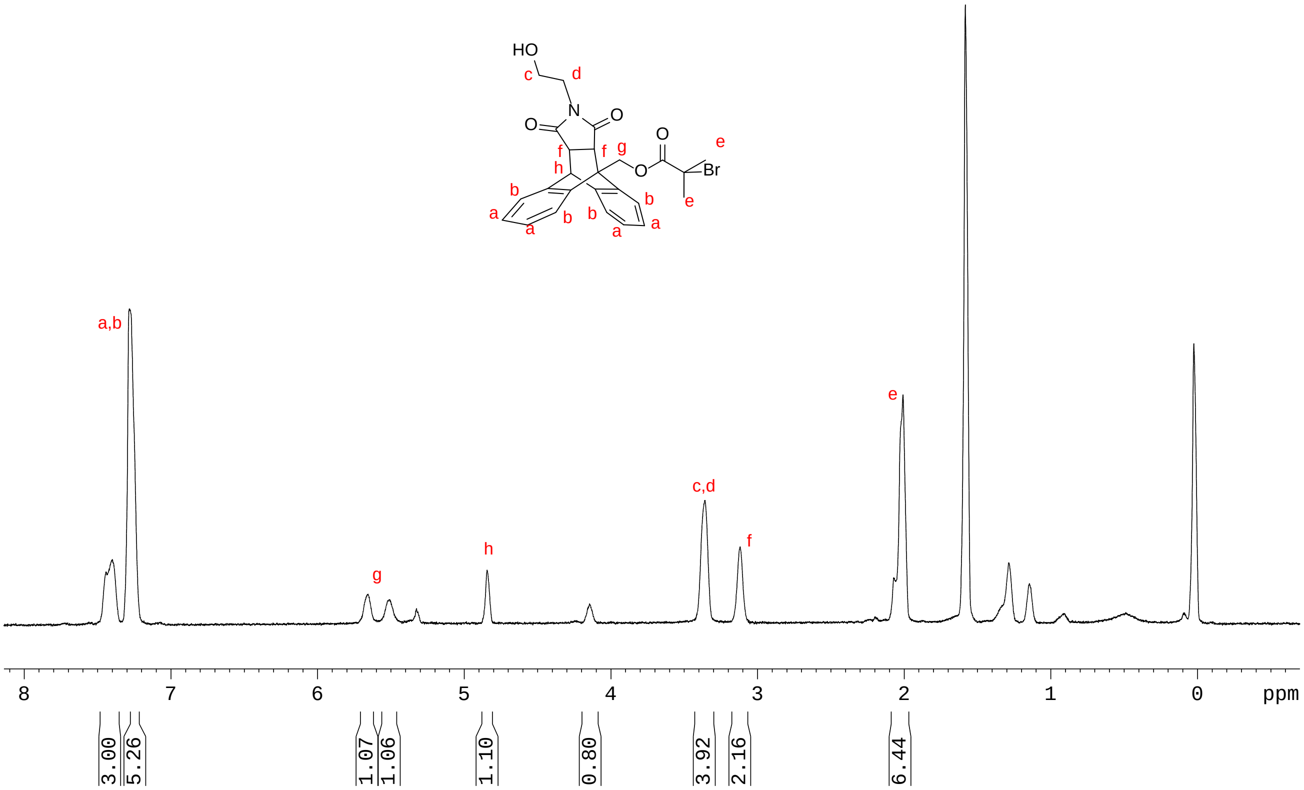

Figure S5. ^{1}H NMR (500 M) spectrum of compound **4** ($CDCl_3$, 7.26 ppm).

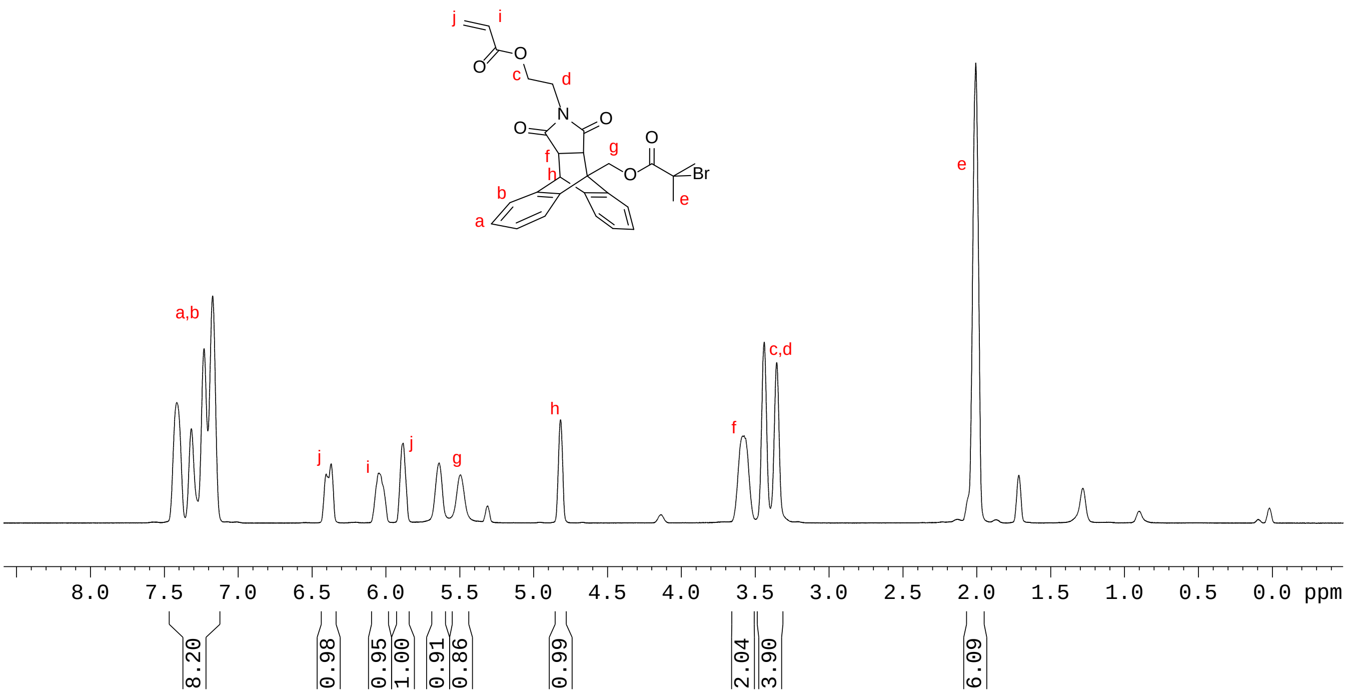

Figure S6. ^{1}H NMR (500 M) spectrum of compound **M0** ($CDCl_3$, 7.26 ppm).

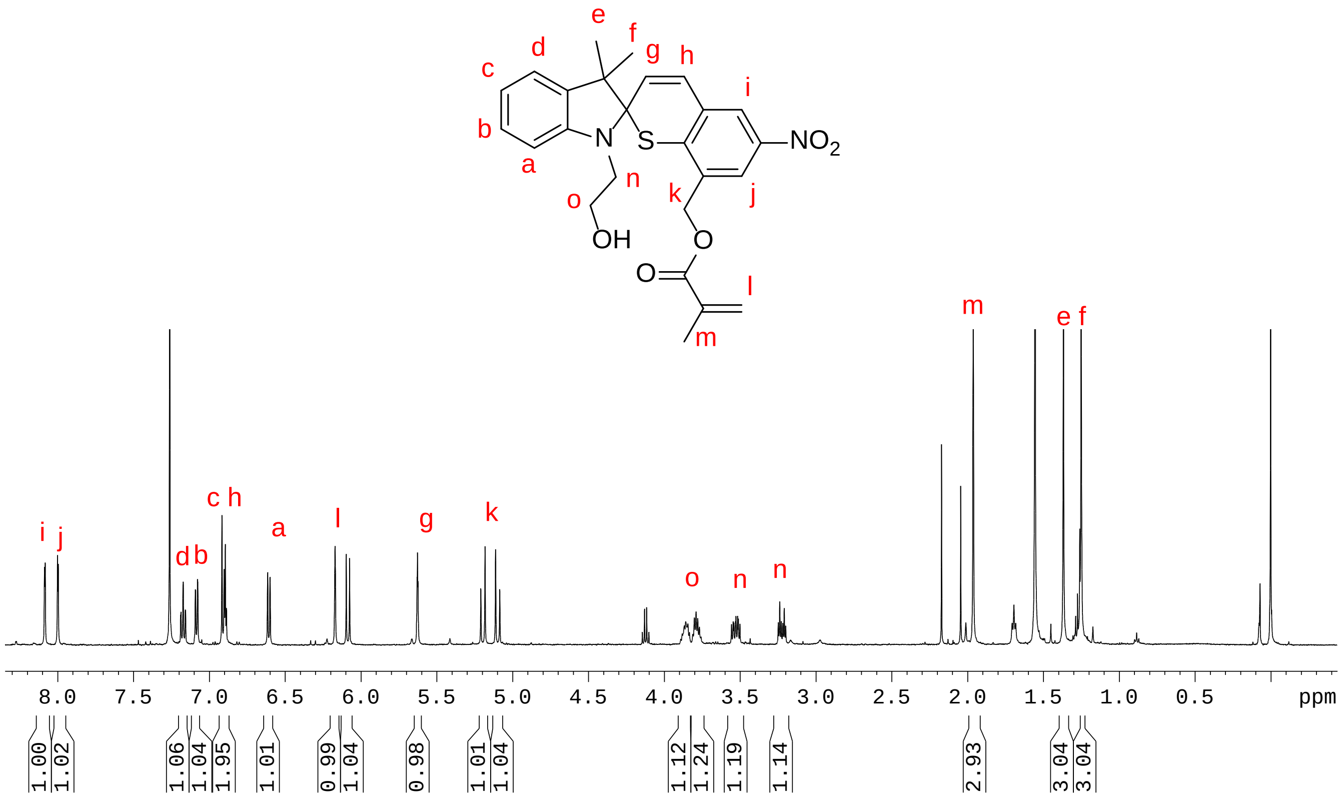

Figure S7. ^{1}H NMR (500 M) spectrum of compound **7** ($CDCl_3$, 7.26 ppm).

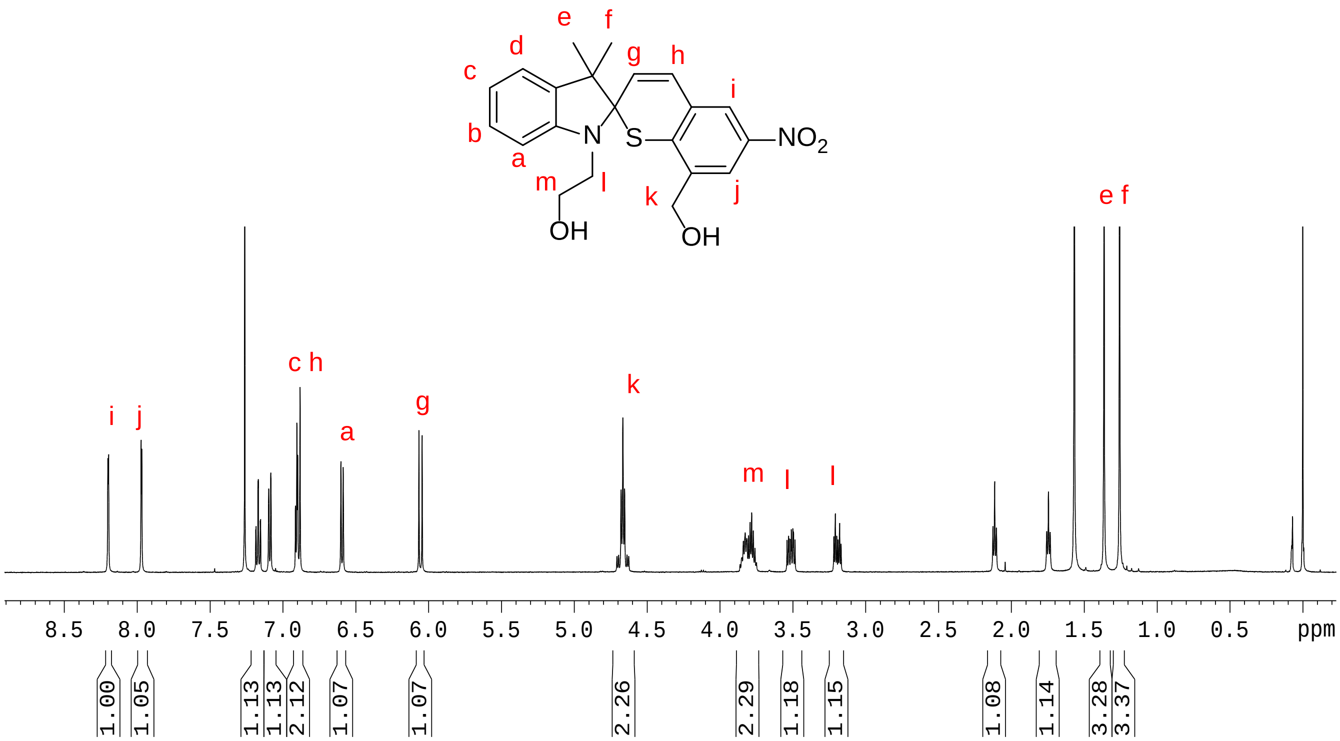

Figure S8. ^{1}H NMR (500 M) spectrum of compound **8** ($CDCl_3$, 7.26 ppm).

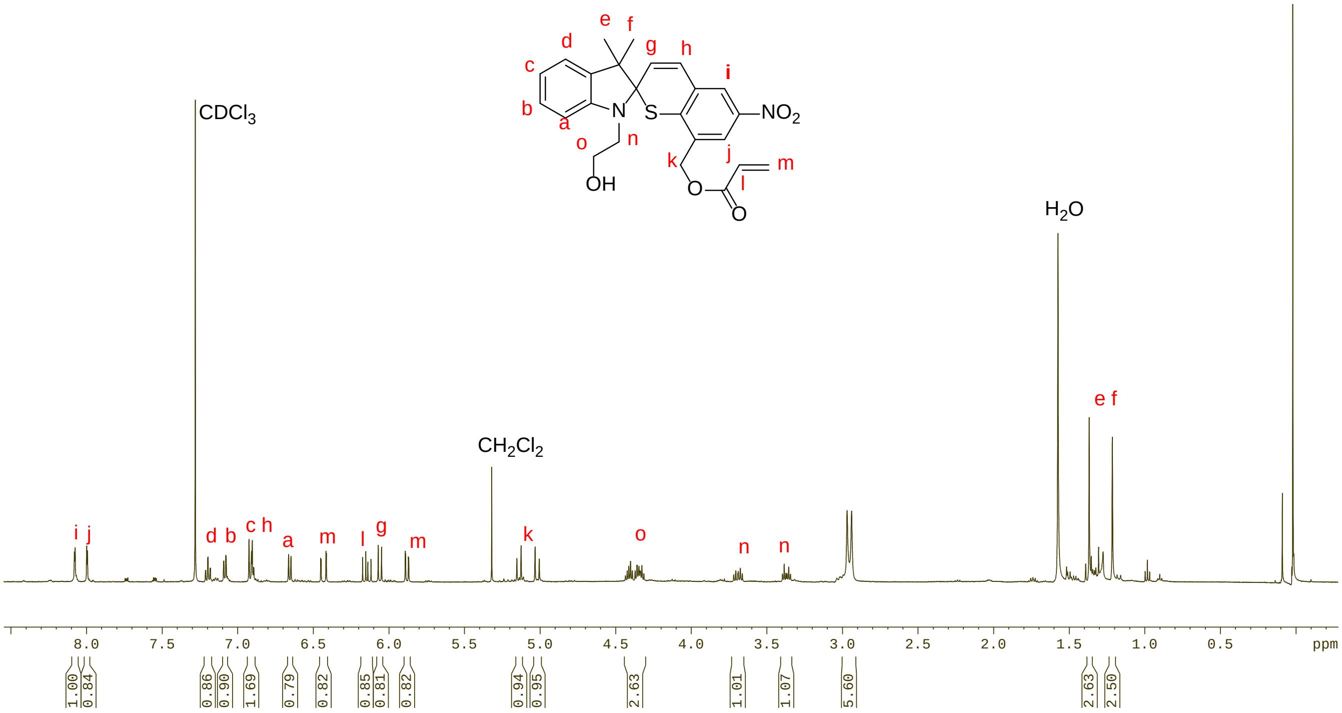

Figure S9. ^{1}H NMR (500 M) spectrum of compound **9** ($CDCl_3$, 7.26 ppm).

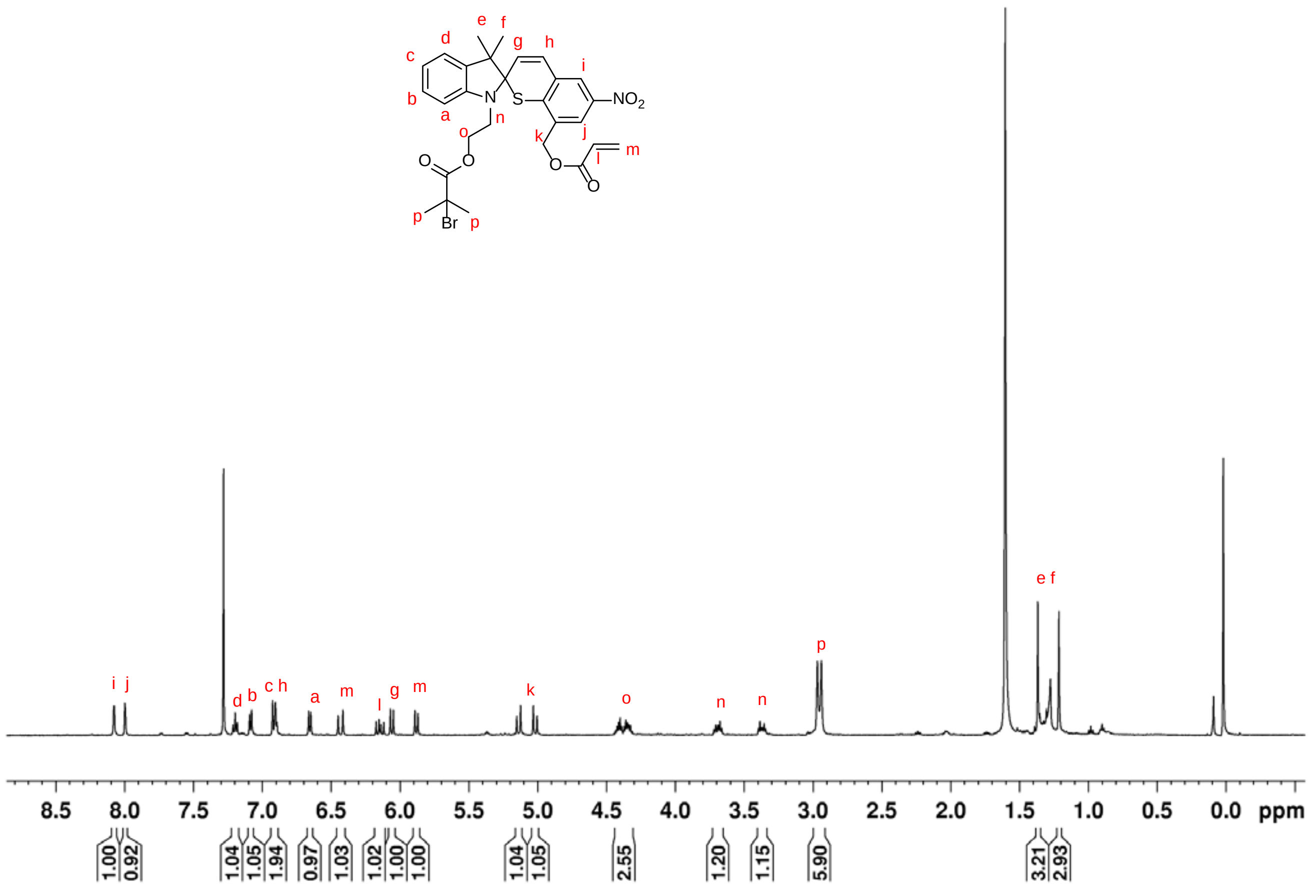

Figure S10. ^{1}H NMR (500 M) spectrum of compound **S0** ($CDCl_3$, 7.26 ppm).

IV. SEC Traces of Polymer M2 and S2

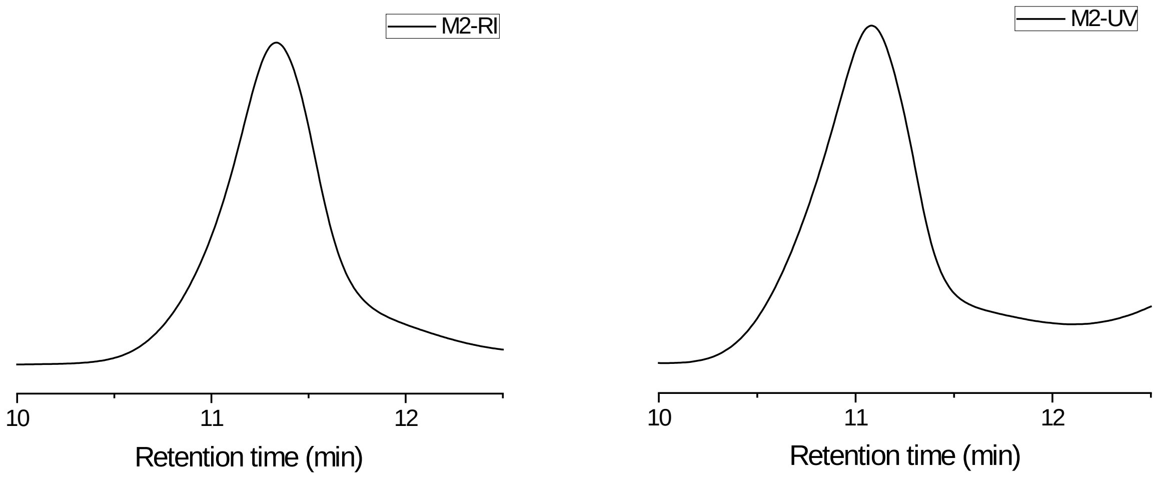

Figure S11. RI (left) and UV-vis (right) output of a SEC of **M2**.

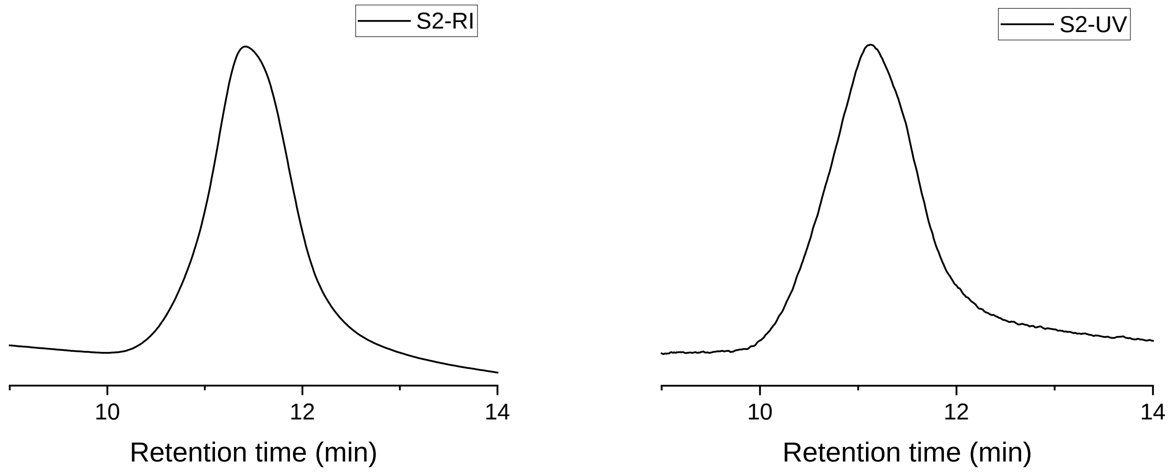

Figure S12. RI (left) and UV-vis (right) output of a SEC of **S2**.

V. Determination of Molar Masses of Polymers

The absence of MALS detector in our SEC setup necessitated the use of Mark-Houwink calibration[9] to estimate molar masses of **M1** and **S1** and additionally the reported values of hydrodynamic contraction to estimate the masses of comb polymers **M2** and **S2**. The Mark-Houwink equation establishes the relationship between the masses of two linear polymers of different composition eluting at the same elution volume of SEC. If one of the two polymers is a standard of known molar mass (e.g., polystyrene, PS), the accurate mass of the other polymer is available by eq. 1 if both pairs of Mark-Houwink parameters (a and K) are known. For both linear polystyrenes (standards) and polyacrylates, multiple Mark-Houwink parameters, covering a range of eluants and temperatures, have been reported.

$$KM^{1+a} = K_{\mathrm{PS}} M_{\mathrm{PS}}^{1+a_{\mathrm{PS}}} \qquad (1)$$

If the nominal molecular mass distribution (MMD) of the analyte in PS mass-equivalents is adequately approximated by a Gaussian function, eq. 1 can be readily extended to the moments of the MMD (e.g., M_n and M_w) because the expectation value of the independent variable (in this case, chain mass) to the power of α, M^{α} (α>0), which for integer α is the αth moments of the distribution (i.e., M_n, M_w, M_z, etc.) is a hypergeometric function of α, M_n and M_w. The latter can be approximated by simple polynomials for narrow ranges of α. In eqs. 2, $M_{n,n}$ and $M_{w,n}$ are nominal number- and weight-average molars masses (i.e., the 1st and 2nd moments of nominal MMD) in PS mass-equivalents, whereas M_n and M_w are true values; and $p = (1+a_{PS})/(1+a)$. The form of eqs. 2 reflects the approximations of the hypergeometric function for p~1 (depending on the side-chains of poly(alkyl acrylates), the measurement temperature and accounting for variation of the reported Mark-Houwink parameters for identical conditions, literature values of a and a_{PS} in THF correspond to $0.93<p<1.01$, Table S1). Note that eqs. 2 reduce to eq. 1 for $a_{PS} = a$ (p=1), as expected.

$$M_{\mathrm{n}} = \left(\frac{K_{\mathrm{PS}}}{K}\right)^{\frac{1}{1+a}} M_{\mathrm{n,n}}^{p} \left(1 + (p-1)\left(\left(\frac{M_{\mathrm{w,n}}}{M_{\mathrm{n,n}}}\right)^{2} - 1\right)\right)^{1/p} \quad \text{(2a)}$$

$$M_{\mathrm{w}} = \left(\frac{K_{\mathrm{PS}}}{K}\right)^{\frac{1}{1+a}} M_{\mathrm{n,n}}^{p} \left(\frac{M_{\mathrm{w,n}}}{M_{\mathrm{n,n}}}\right)^{\frac{1}{p}} \left(1 + 4(p-1)\left(1 - \left(\frac{M_{\mathrm{n,n}}}{M_{\mathrm{w,n}}}\right)^{2}\right)\right)^{1/2p} \quad \text{(2b)}$$

Because the nominal MMDs of **M2** and **S2** are close to Gaussian, we used eqs. 2 to convert nominal moments, $M_{\mathrm{n,n}}$ and $M_{\mathrm{w,n}}$ to true moments, M_{n}, and M_{w}. The presence of Br atom in every side chain of **M1** and **S1** requires a multiplier applied to the values estimated by eqs. 2, because such heavy atoms increase the chain mass without changing its hydrodynamic radius and thus the elution volume.[10] The multipliers are 1.17 for **M1** and 1.02 for **S1**. The smaller value for **S1** reflects both the higher molar mass of **S0** (monomer) and the smaller fraction of **S0** in **S1** as reflected by the monomer feed ratio in polymerization of **S1**. The high density of bulky side groups in **M1** prompted us to use the reported Mark-Houwink parameters (a and K in eqs. 1-2) for poly(t-butyl acrylate) for **M1** but PMA for **M2**, **S1** and **S2**.

Accurate evaluation of molar masses of comb polymers **M2** and **S2** present an additional challenge in that a branched polymer has a smaller hydrodynamic radii than a linear polymer of the same composition and molar mass in the same solvent, a phenomenon called contraction.[11] As a result, the mass of a branched polymer, M_{B}, eluting at the same volume, V (or the corresponding retention time) as a linear polymer of the same composition will be greater than that of the linear polymer, M_{L}. The ratio of the two masses at the same elution volume, g' (eq. 3) is independent of the elution volume (a is the Mark-Houwink exponent of the linear polymer). As a result, the moments of the mass distribution of a branched polymer, $M_{\mathrm{n,B}}$ and $M_{\mathrm{w,B}}$ are proportional to the moments of the distribution of the linear polymer of the same composition, $M_{\mathrm{n,L}}$ and $M_{\mathrm{w,L}}$ (eqs. 4). The true linear-equivalent masses, $M_{\mathrm{n,L}}$ and $M_{\mathrm{w,L}}$ are estimated by eqs. 2 from experimentally measured nominal moments.

$$g' = \left(\frac{M_{\mathrm{L}}}{M_{\mathrm{B}}}\right)_{V}^{1+a} \quad \text{(3)}$$

$$M_{\mathrm{n,B}} = \frac{M_{\mathrm{n,L}}}{g'^{\frac{1}{1+a}}} \quad \text{(4a) and} \quad M_{\mathrm{w,B}} = \frac{M_{\mathrm{w,L}}}{g'^{\frac{1}{1+a}}} \quad \text{(4b)}$$

The value of *g*' depends strongly on the topology of the polymer (e.g., star, brush, comb, etc) and branching ratio but only weakly on polymer composition in good solvents.[12] For comb polymers, *g*' decreases rapidly with branching density when the average separation of branching points along the backbone is larger or comparable to the average length of the side chain but it levels of at ~0.58 (in a good solvent) when the this separation is much smaller than the side-chain length. The ratios of the nominal masses, $M_{\mathrm{n,n}}$, of **M1** to **M2** and **S1** to **S2** (~60 and ~20, respectively) suggest that the side chains of **M2** and **S2** are considerably longer than the average distance between branching points, justifying the use of *g*' = 0.58 for estimating true M_{n} and M_{w} of **M2** and **S2** (Table S1).

Table S1. Summary of the observed and estimated moments of MMDs of the reported polymers and the values of constants used for the estimates. The errors on masses reflect the uncertainty of the constants and disregard errors on measured (nominal) moments. All masses are in kDa.

	nominal		true						M_n, side chain	
	$M_{n,n}$	$M_{w,n}$	M_n	M_w	$Đ_M$	p	K_{PS}/K		From SEC	From sonication
**M1**	7	11	9.9±1.6	15±3	1.6	0.98±0.02[a]	1.5±0.4[a]	18±3[b]		
**M2**	570	720	1100±120	1400±200	1.3	0.96±0.04[c]	2.0±0.3[c]		60±9[d]	56±4[e]
**S1**	15	24	20±3	34±6	1.7	0.96±0.04[c]	2.0±0.3[c]	15±2[f]		
**S2**	320	520	580±70	980±130	1.7	0.96±0.04[c]	2.0±0.3[c]		38±8[d]	44±5[e]

(a) for poly(t-butyl acrylate), average from [13]

(b) the number of the DA adducts, branch and initiator moieties per average chain of **M1**

(c) for poly(methyl acrylate), average from [13]

(d) average mass of the side chain in an average comb chain

(e) from modeling for **M2** (see above) and from M_n of the low-mass component of the sonicated mixture of **S2** at the end of sonication (Figure S22)

(f) the number of the STP, branch and initiator moieties per average chain of **S1**.

We used *g*'=0.58 and eq. 2a to estimate the changes of true M_n of sonicated solutions of **M2**. This sonication produces either linear anthracene-terminated chains (fragmentation by dissociation of the DA adduct) or comb polymers with the same branching density as **M2**. Because *g*' is identical for chains of any mass of the same topology and branching density, we estimated true M_n by representing the nominal MMD of each sonicated sample as a sum of Gaussians and applying the contraction correction only to Gaussians with M_n > 90 kDa (1.5 times the length of the average side-chain, "chains subject to *g*'" in Table S2 below). The overall true Mn of the sample was taken as fraction weighed some of the Mn's of the "high-mass" (branched polymer, *g*' = 0.58) and "low-mass" (linear polymers, *g*' = 1) components. The relevant numbers are summarized in Table S2 below.

Table S2. Summary of the moments of the MMDs of sonicated solutions of **M2**.

Sonication time	$M_{n,n}$	Chains subject to *g*'			Chains not subject to *g*'		M_n
		fraction	$M_{n,n}$	M_n	$M_{n,n}$	M_n	
0	560	1.0	560	1100			1100
15	187	0.45	310	486	55	75	260
30	102	0.22	280	409	45	73	147
45	91	0.18	250	328	42	70	116
60	76	0.12	235	280	40	68	93
90	60	0.1	230	280	42	63	85
120	49	0.06	220	270	40	58	71

VI. Mechanochemical Activation of the DA adduct and STP Moieties

General sonication procedures, SEC, UV-Vis and NMR analysis

Ultrasound experiments were performed on a Vibra Cell 505 liquid processor with a 12.8 mm (diameter) titanium solid probe (Sonics and Materials). For a typical sonication experiment, polymers were dissolved in 8 mL THF or DMF to the concentration of 5 mg/mL. The solution was then transferred to a 3-necked cell in an ice bath and sparged with N_2 for 20 min. A pulse sequence of 1s on/1s off was applied to the solution at a power of 8.7 W/cm^2. The temperature of the system was maintained at 0 - 5 ^{o}C. The sonication was carried out under N_2.

Aliquots (0.5 mL) at given times were removed from the cell for SEC and UV-Vis tests. For SEC measurements the aliquots were diluted to 0.8 mg/mL. To monitor the thiol-ene addition reaction of STP with NEM, UV-vis spectra were recorded. For ^{1}H NMR measurement, methanol was added to the aliquots to precipitate the sonicated polymer, which was filtered, washed with methanol and redissolved in $CDCl_3$ (~25 mg/mL) for further analysis.

Mechanochemical activation of DA moieties in M1 and M2

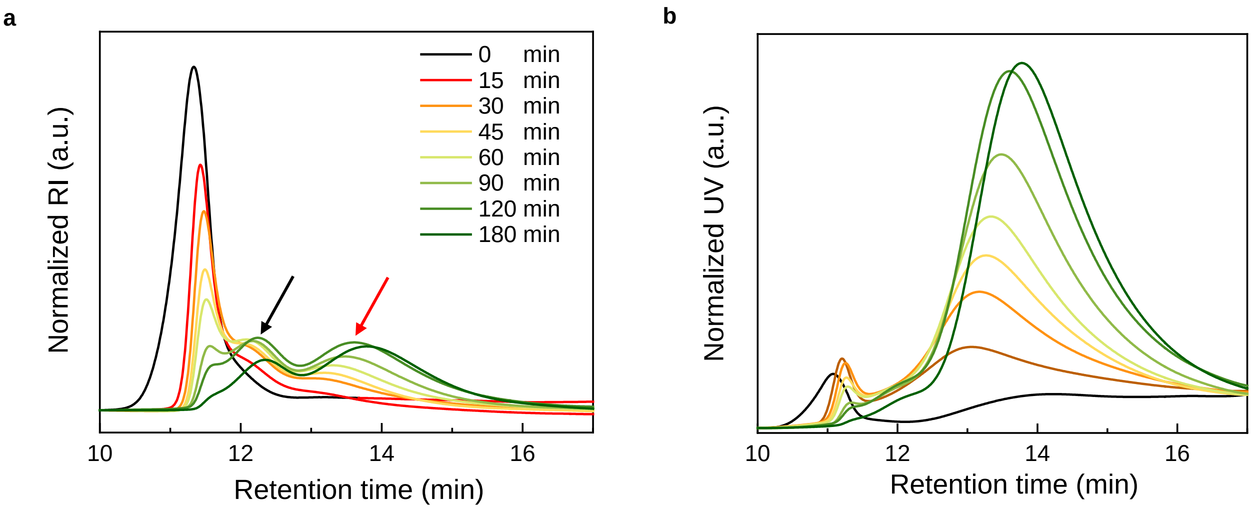

Figure S13. Summary of the mechanochemical retro-DA reaction of comb polymer **M2** in solution (5 mg/mL, THF, 1 s on and 1 s off). SEC traces of **M2** as a function of ultrasonication time (a: RI, b: UV). The black and red arrow indicate peaks corresponding to half of the starting polymer and to free side chains, respectively.

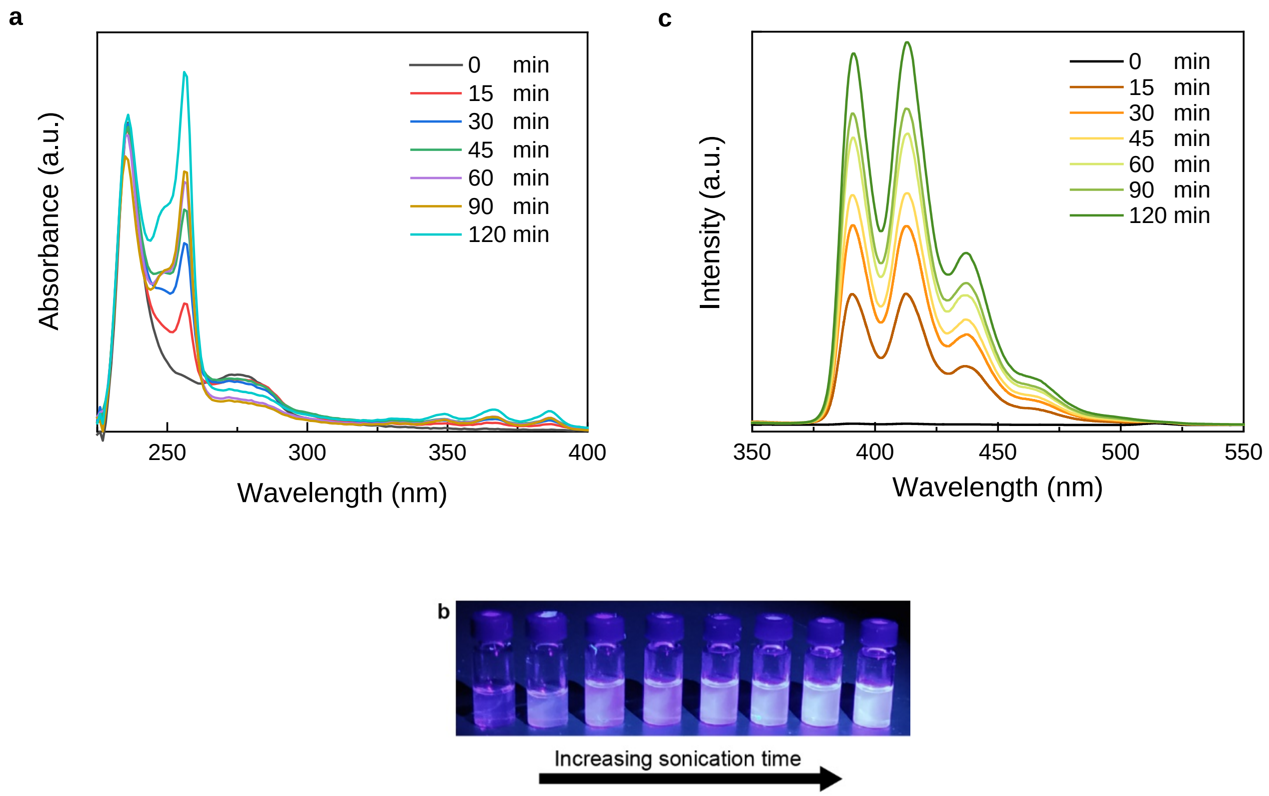

Figure S14. UV-Vis spectra (a), fluorescence images (b) and spectra (c) (256 nm excitation) of **M2** (aliquots diluted to 1 mg/mL) with increasing sonication time.

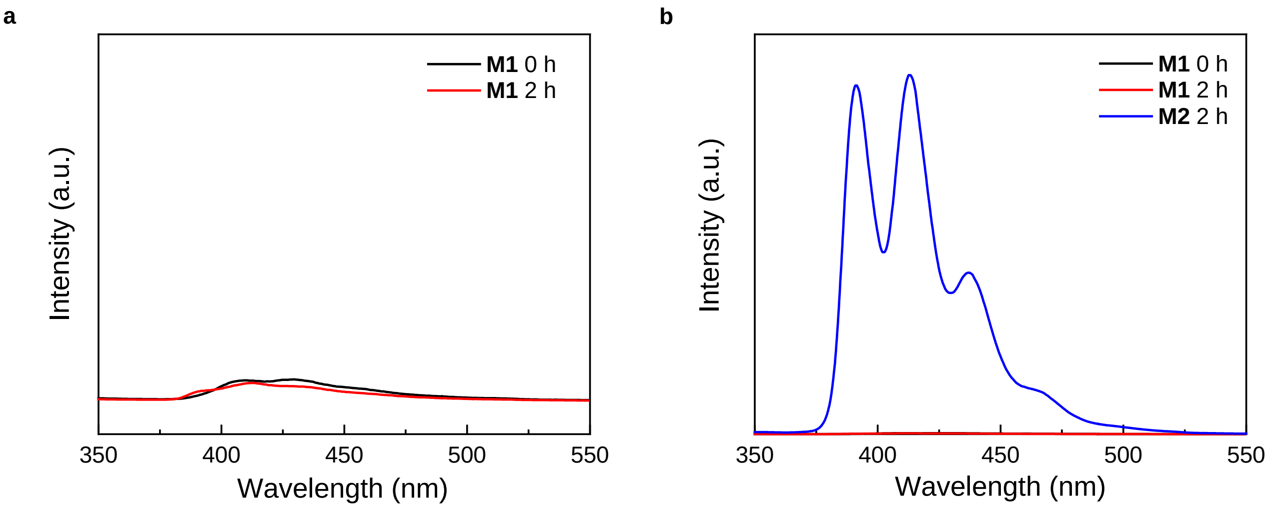

Figure S15. Fluorescence spectra of **M1** before and after 2 h sonication under identical conditions as **M2** (5 mg/mL, THF, 10 W/cm^2, 0 - 5 ^{o}C, 1 s on and 1 s off). Aliquots used for fluorescence spectropy were diluted to 1 mg/mL.

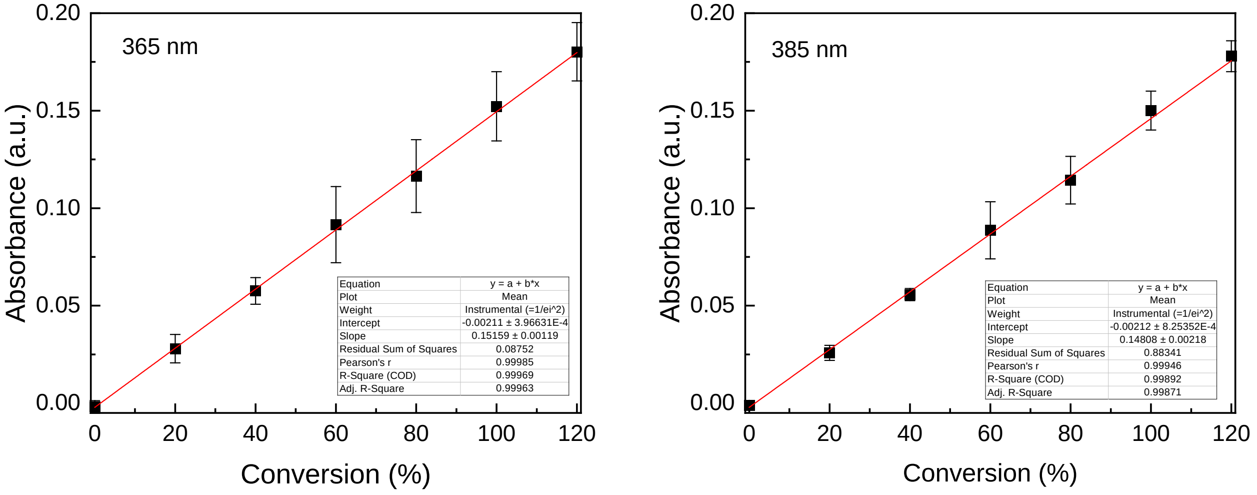

Figure S16. Calibration cures relating the absorbance of 9-anthracene methanol in THF at 365 nm and 385 nm to the theoretical conversion (fraction of dissociation) of anthracene/maleimide in **M2**.

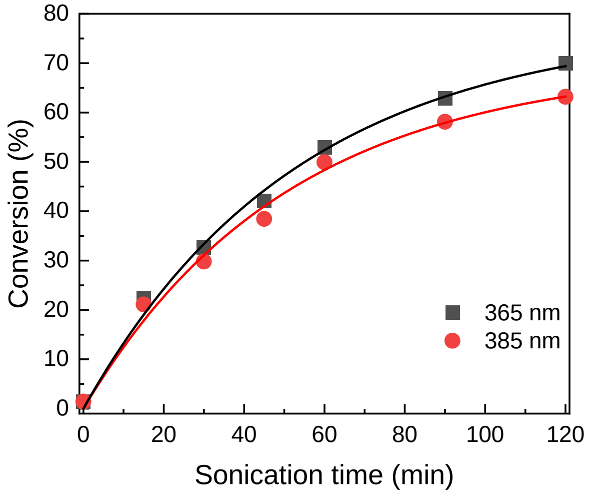

Figure S17. Calculated mechanochemical conversion (fraction of dissociation) of anthracene/maleimide in **M2** as function of sonication time using the calibration curves in Figure S16. Rate constants were fitted using the conversion data based on the method described below.

According to Figure S17, the fitting using the conversion data from different wavelengths gave similar values of the rate constant:

Wavelength (nm)	365	385
Rate constant (min^{-1})	0.0188	0.0196

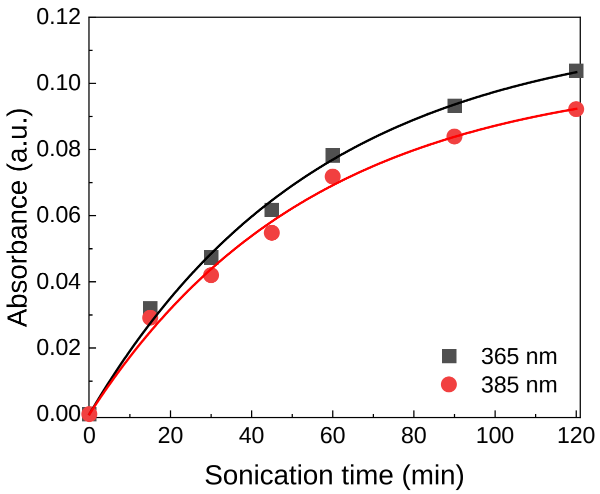

Figure S18. Rate constants of the conversion of anthracene/maleimide in **M2** were also obtained by directly fitting the absorbance based on the method described below.

According to Figure S18, the fitting using absorbance data from different wavelengths gave similar values of the rate constant:

Wavelength (nm)	365	385
Rate constant (min^{-1})	0.0178	0.0183

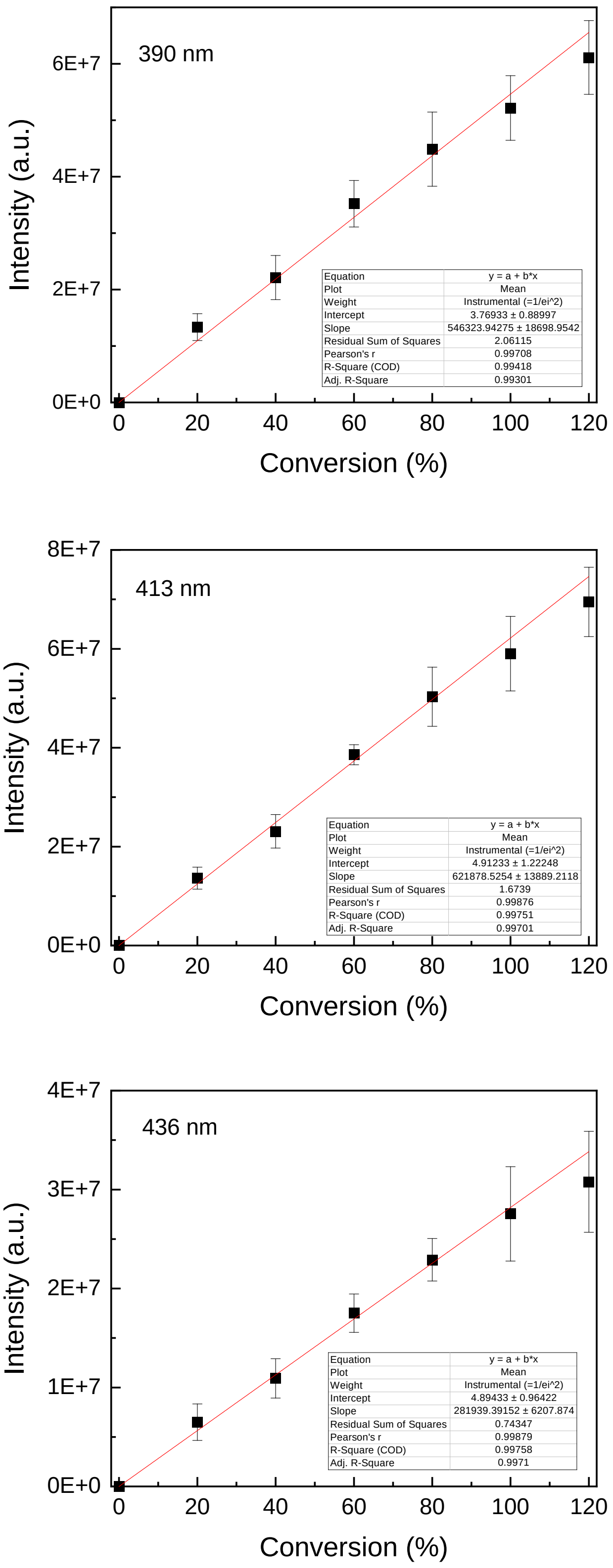

Figure S19. Calibration cures relating the fluorescence intensity of 9-anthracene methanol in THF at 390 nm, 413 nm and 436 nm to the theoretical conversion (fraction of dissociation) of anthracene/maleimide in **M2**.

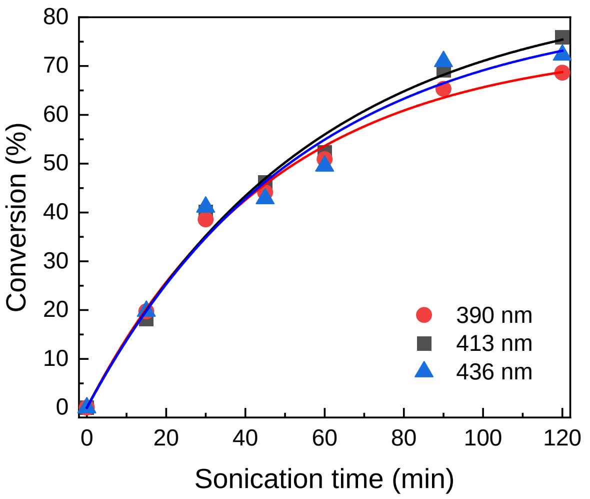

Figure S20. Calculated mechanochemical conversion (fraction of dissociation) of anthracene/maleimide in **M2** as function of sonication time based on the calibration curves in Figure S19. Rate constants were obtained by fitting using the conversion data based on the method described below.

According to Figure S20, the fitting using conversion data from different wavelengths gave similar values of the rate constant, which are also similar to those obtained from UV-Vis measurements:

Wavelength (nm)	390	413	436
Rate constant (min^{-1})	0.0211	0.0176	0.0184

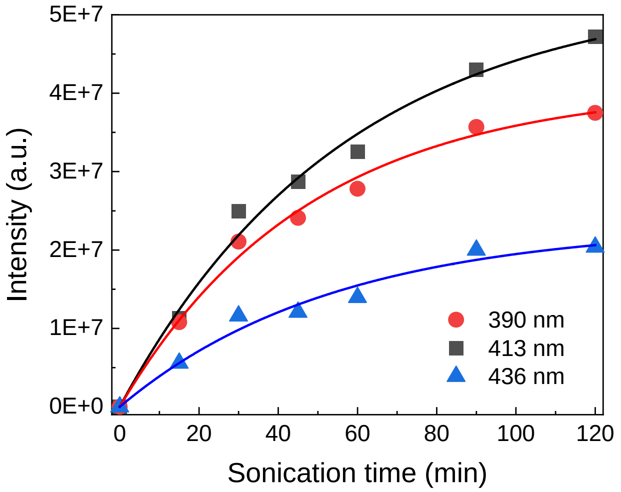

Figure S21. Rate constants were also obtained by directly fitting the fluorescence intensity data at three different wavelengths based on the method described below.

According to Figure S21, the fitting using fluorescent intensity data from different wavelengths gave similar values of the rate constant, which are also similar to those obtained from UV-Vis measurements:

Wavelength (nm)	390	413	436
Rate constant (min^{-1})	0.0211	0.0176	0.0184

Estimation of reaction rates

The retro-DA reaction of anthracene/maleimide adduct in **M2** during ultrasound sonication may be represented as:

$$\mathrm{An-Mal} \xrightarrow{k} \mathrm{An+Mal}$$

where An-Mal represents the anthracene/maleimide moieties in comb polymer **M2**, An is the observed anthracenyl portion, and Mal is the maleimide portion of the sonicated product.

The rate of the retro-DA reaction is assumed to fulfill the first-order kinetics:

$$-\frac{d[\mathrm{An-Mal}]}{dt} = k[\mathrm{An}] \qquad (5)$$

Solving Equation (2) in terms of the concentration of An yields:

$$[\mathrm{An}] = [\mathrm{An}]_\infty(1 - e^{-kt}) \qquad (6)$$

where $[\mathrm{An}]_\infty$ is the concentration of An at infinite time (may not necessarily equals to the concentration of DA).

The fluorescent intensity/absorbance of the postsonicated solution, as measured by fluorescence spectroscopy/UV-vis spectroscopy, is directly related to the concentration of the anthracenyl species by:

$$I_f = mI_0\phi[1 - 10^{-\varepsilon bc}] \qquad (7)$$

where

m is a proportionality constant attribute to the instrument

I_0 is the incident light intensity

ε is the molar absorptivity

Φ is the quantum yield

c is the concentration, and

b is the path length

in dilute solution where $10x \approx 1 + x$, eq. 7 can be approximated to:

$$I_f = mI_0\phi[\varepsilon bc] \quad (8)$$

substituting eq. 8 into eq. 6, we then have

$$I_f = I_{f\infty}(1 - e^{-kt}) \quad (9)$$

Hence fitting the fluorescence intensity/absorbance of the post-sonicated **M2** solution will yield the rate of the retro-DA reaction, k.

Mechanical and photochemical activation of STP in S2

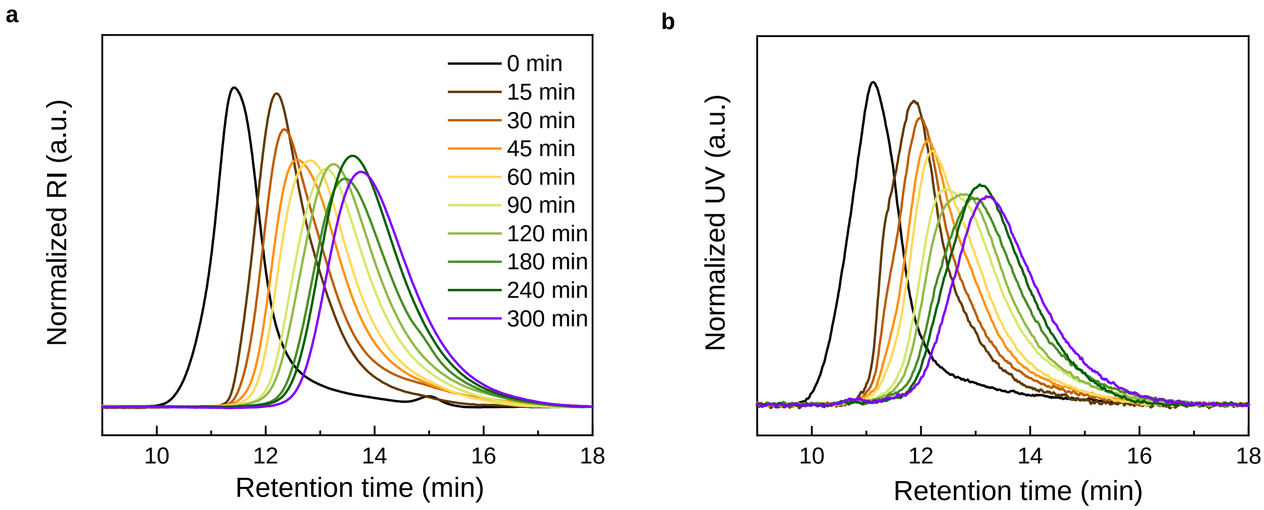

Figure S22. Mechanochemical activation of STP in **S2** in solution (5 mg/mL, DMF, 1 s on and 1 s off). SEC traces (a: RI, b: UV) of **S2** as a function of ultrasonication time.

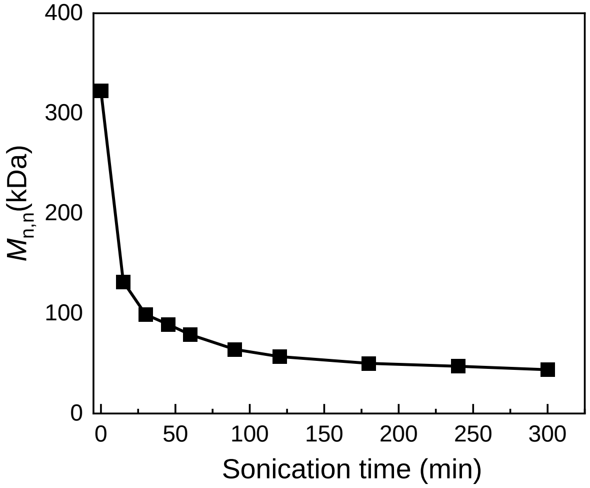

Figure S23. Non-selective degradation of **S2** in ultrasound sonication. Decrease of the nominal number-averaged molecular weight, $M_{n,n}$ (in PS mass equivalents) as a function of time.

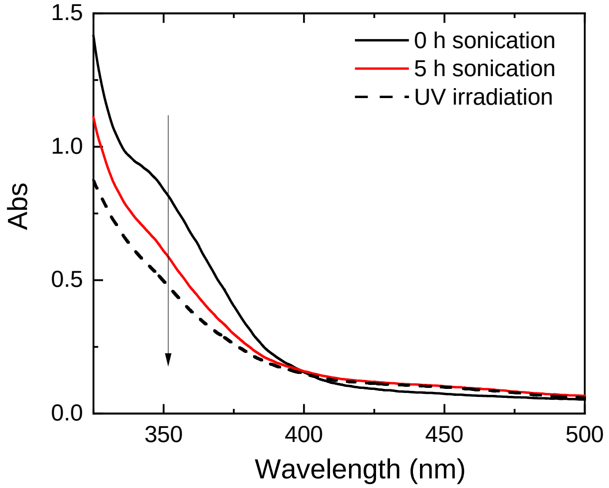

Figure S24. UV/Vis spectra of **S2** before and after sonication in the presence of N-ethylmaleimide (NEM) (STP: NEM = 1:1.5) (black and red) and after UV irradiation (365 nm, black dashed line) in the presence of NEM (STP: NEM = 1:1.5) with no sonication (5 mg/mL, THF, 10 W/cm^2, 0 - 5 ^{o}C, 1 s on and 1 s off). The arrow indicate the drop of the absorbance at 350 nm of STP.

The characteristic band of STP at 365 (nm) decreased because of the thiol-ene addition between mechanochemically generated TMC and the added NEM.[7] Similar UV/Vis profile was also observed upon irradiation of the same solution at 365 nm.

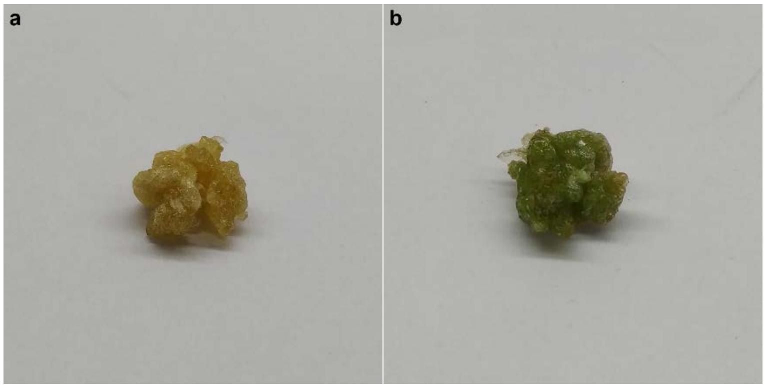

Figure S25. Activation of bulk **S2** by 365 nm UV light: (a) before irradiation; (b) after irradiation of 5 min. The color is fully reversible when kept in the dark or irradiated using white light, indicating reversible isomerization between STP and TMC.

VII. Load-Induced Crosslinking between M2 and S2

Load-triggered crosslinking in solution

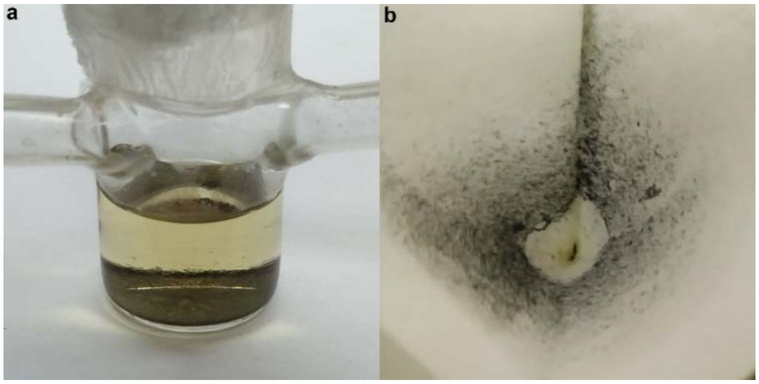

Figure S26. Mechanochemical cross-linking of polymers **M2** and **S2** in sonicated THF solution. The precipitates after 12 h resting (a) and after filtration (b).

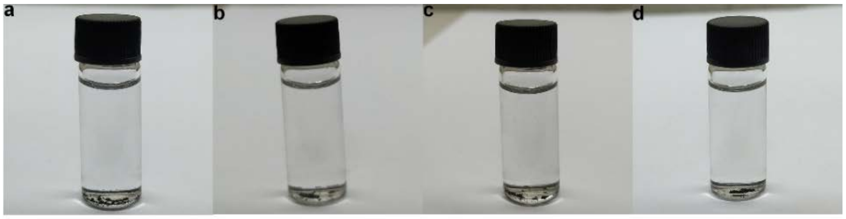

Figure S27. The dark green precipitates induced by ultrasound sonication of **M2** + **S2** are insoluble in (a) THF, (b) DCM, (c) DMF, and DMF (70 ^{o}C).

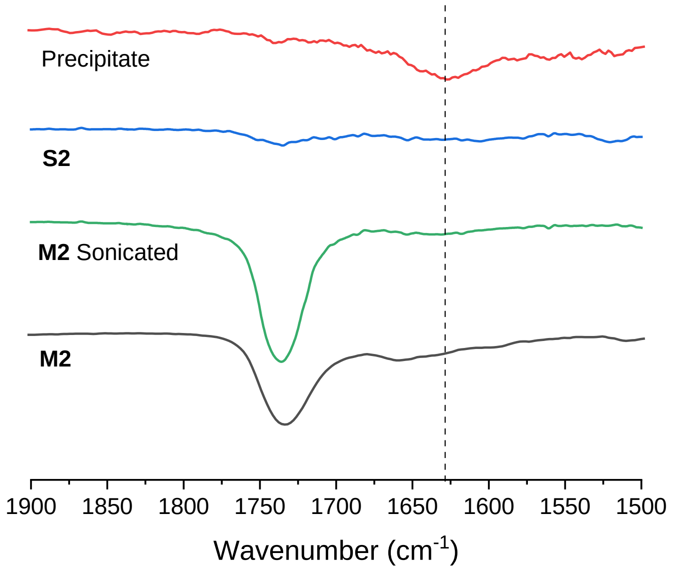

Figure S28. Changes in the absorbance of the C=O stretch of an amide. According to calculations at the CAM-B3LYP/6-31+G(d) level, dissociation of the DA adduct is associated with a negligible (7 cm^{-1}) shift of the C=O band of maleimide. In contrast, the addition of TMC to maleimide causes the C=O stretch to shift to a lower frequency by 167 cm^{-1}. These calculations agree qualitatively with the observed changes in the IR spectra. S2 doesn't contain maleimide and hence doesn't manifest the stretch.

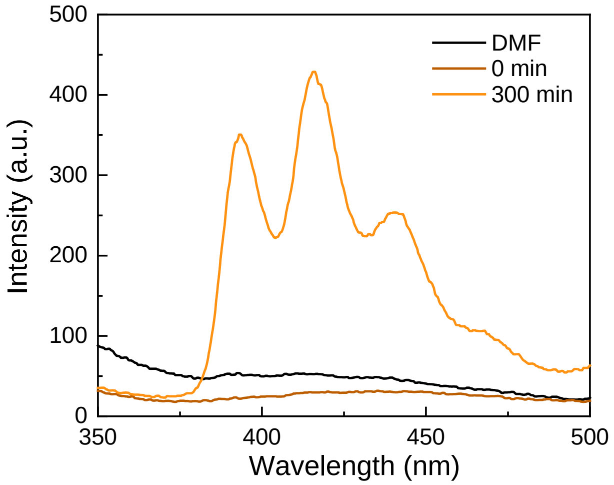

Figure S29. The corresponding fluorescence spectra (256 nm excitation) of the mixture of **M2** + **S2** (i) before and (ii) after 300 min sonication. Aliquots were diluted to 1 mg/mL.

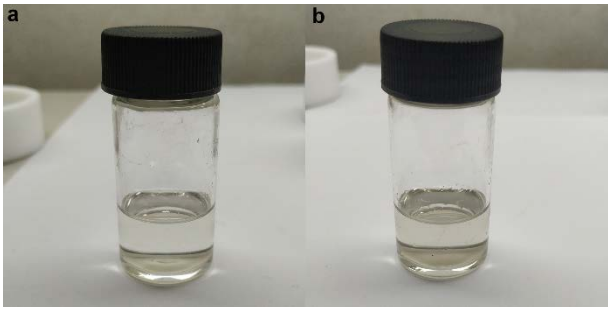

Figure S30. Sonicating a mixture of **S2** (5 mg/mL) and **M2** (20 mg/mL) in DMF: (a) before sonication; (b) after sonication.

Reducing the concentration of **S2** from 50 mg/mL to 5 mg/mL yielded no precipitation, i.e., no chain cross-linking.

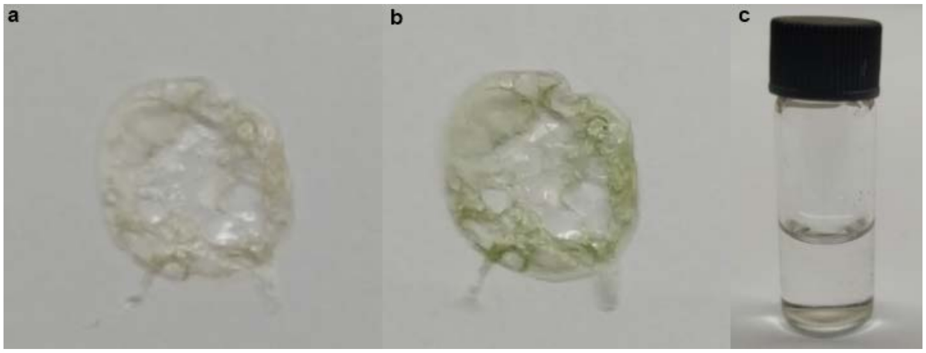

Figure S31. (a) Film made of mixture of **S2** and **M2**. (b) The film was irradiated under UV light 365 nm, 20 min. (c) The mixture is fully soluble in DCM.

A film made of the mixture of **M2** and **S2** was irradiated by UV light. The colour of the film turned into green (TMC), however, the color is fully reversible and he resulting film can be fully redissolved in DCM, suggesting no formation of chain cross-linking when the DA adduct in **M2** was not activated.

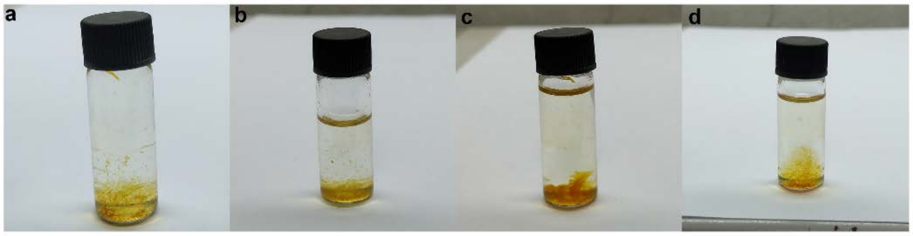

Figure S32. The solids formed by UV irradiation of the mixture of sonicated **M2** and virgin **S2** in bulk are insoluble in (a) THF, (b) DCM, (c) DMF, and DMF (70 ^{o}C).

VIII. References

[1] M. J. Frisch, G. W. Trucks, H. B. Schlegel, G. E. Scuseria, M. A. Robb, J. R. Cheeseman, G. Scalmani, V. Barone, B. Mennucci, G. A. Petersson, H. Nakatsuji, M. Caricato, X. Li, H. P. Hratchian, A. F. Izmaylov, J. Bloino, G. Zheng, J. L. Sonnenberg, M. Hada, M. Ehara, K. Toyota, R. Fukuda, J. Hasegawa, M. Ishida, T. Nakajima, Y. Honda, O. Kitao, H. Nakai, T. Vreven, J. J. A. Montgomery, J. E. Peralta, F. Ogliaro, M. Bearpark, J. J. Heyd, E. Brothers, K. N. Kudin, V. N. Staroverov, R. Kobayashi, J. Normand, K. Raghavachari, A. Rendell, J. C. Burant, S. S. Iyengar, J. Tomasi, M. Cossi, N. Rega, J. M. Millam, M. Klene, J. E. Knox, J. B. Cross, V. Bakken, C. Adamo, J. Jaramillo, R. Gomperts, R. E. Stratmann, O. Yazyev, A. J. Austin, R. Cammi, C. Pomelli, J. W. Ochterski, R. L. Martin, K. Morokuma, V. G. Zakrzewski, G. A. Voth, P. Salvador, J. J. Dannenberg, S. Dapprich, A. D. Daniels, Ö. Farkas, J. B. Foresman, J. V. Ortiz, J. Cioslowski, D. J. Fox, **2009**.

[2] C. J. Cramer, *Essentials of Computational Chemistry*, 2nd ed. ed., Wiley, Chichester, **2004**.

[3] T. J. Kucharski, R. Boulatov, *J. Mater. Chem.* **2011**, *21*, 8237-8255.

[4] G. S. Kochhar, G. S. Heverly-Coulson, N. J. Mosey, in *Polymer Mechanochemistry, Vol. 369* (Ed.: R. Boulatov), Springer International Publishing, Cham, **2015**, pp. 37-96.

[5] Y. Tian, R. Boulatov, *ChemPhysChem* **2012**, *13*, 2277-2281.

[6] S. Akbulatov, Y. Tian, Z. Huang, T. J. Kucharski, Q.-Z. Yang, R. Boulatov, *Science* **2017**, *357*, 299-303.

[7] H. Zhang, F. Gao, X. Cao, Y. Li, Y. Xu, W. Weng, R. Boulatov, *Angew. Chem. Int. Ed.* **2016**, *55*, 3040-3044.

[8] T. Gruendling, S. Weidner, J. Falkenhagen, C. Barner-Kowollik, *Polym. Chem.* **2010**, *1*, 599-617.

[9] in *Modern Size‐Exclusion Liquid Chromatography*, **2009**, pp. 193-229.

[10] M. Schaefer, B. Icli, C. Weder, M. Lattuada, A. F. M. Kilbinger, Y. C. Simon, *Macromolecules* **2016**, *49*, 1630-1636.

[11] W. Burchard, in *Branched Polymers II* (Ed.: J. Roovers), Springer Berlin Heidelberg, Berlin, Heidelberg, **1999**, pp. 113-194.

[12] S. Lee, T. Chang, *Macromol. Chem. Phys.* **2017**, *218*, n/a.

[13] T. Gruendling, T. Junkers, M. Guilhaus, C. Barner-Kowollik, *M Macromol. Chem. Phys.* **2010**, *211*, 520-528.
